# Sua5 catalyzing universal t^6^A tRNA modification is responsible for multifaceted functions of the KEOPS complex in *Cryptococcus neoformans*

**DOI:** 10.1128/msphere.00557-23

**Published:** 2023-12-12

**Authors:** Yeseul Choi, Hana Hyeon, Kangseok Lee, Yong-Sun Bahn

**Affiliations:** 1 Department of Biotechnology, College of Life Science and Biotechnology, Yonsei University, Seoul, South Korea; 2 Department of Life Science, Chung-Ang University, Seoul, South Korea; University of Georgia, Athens, Georgia, USA

**Keywords:** t^6^A tRNA modification, Sua5, Bud32, Kae1, Qri7, fungal meningitis

## Abstract

The N^6^-threonylcarbamoyl adenosine (t^6^A) tRNA modification is critical for ensuring translation fidelity across three domains of life. Our prior work highlighted the KEOPS complex, organized in a Pcc1-Kae1-Bud32-Cgi121 linear arrangement, not only serves an evolutionarily conserved role in t^6^A tRNA modification but also exerts diverse functional impacts on pathobiological characteristics in *Cryptococcus neoformans*, a leading cause of fungal meningitis worldwide. However, the extent to which the pleiotropic functions of the KEOPS complex are specifically tied to tRNA modification remains uncertain. To address this, we undertook a functional characterization of Sua5, responsible for generating the precursor threonylcarbamoyl-adenylate (TC-AMP) for t^6^A tRNA modification, using a reverse genetics approach. Comparative phenotypic analyses with KEOPS mutants revealed that Sua5 plays a vital role in multiple cellular processes, such as t^6^A tRNA modification, growth, sexual development, stress response, and virulence factor production, thus reflecting the multifaceted functions of the KEOPS complex. In support of this, *sua5*Δ *bud32*Δ double mutants showed phenotypes comparable to those of the corresponding single mutants. Intriguingly, a *SUA5* allele lacking a mitochondria targeting sequence (*SUA5^MTS^*^Δ^) was sufficient to restore the wild-type phenotypes in the *sua5*Δ mutant, suggesting that Sua5’s primary functional locus may be cytosolic, akin to the KEOPS complex. Further supporting this, the deletion of Qri7, a mitochondrial paralog of Kae1, had no discernible phenotypic impact on *C. neoformans*. We concluded that cytosolic t^6^A tRNA modifications, orchestrated by Sua5 and the KEOPS complex, are central to the regulation of diverse pathobiological functions in *C. neoformans*.

**IMPORTANCE:**

Understanding cellular functions at the molecular level is crucial for advancing disease treatments. Our research reveals a critical connection between the KEOPS complex and Sua5 in *Cryptococcus neoformans*, a significant cause of fungal meningitis. While the KEOPS complex is known for its versatile roles in cellular processes, Sua5 is specialized in t^6^A tRNA modification. Our key finding is that the diverse roles of the KEOPS complex, ranging from cell growth and stress response to virulence, are fundamentally linked to its function in t^6^A tRNA modification. This conclusion is supported by the remarkable similarities between the impacts of Sua5 and KEOPS on these processes, despite their roles in different steps of the t^6^A modification pathway. This newfound understanding deepens our insight into fungal biology and opens new avenues for developing potential therapies against dangerous fungal diseases.

## INTRODUCTION

Transfer RNA (tRNA) has a significant role in transferring amino acids for translation, encoded from the mRNA genetic code, which is a key biological process in all living organisms (1). Structurally, tRNA comprises 70–100 nucleotides and forms a unique tertiary cloverleaf-like structure through interactions between different regions of the RNA molecule. Each tRNA molecule carries a specific amino acid to pair with the corresponding anticodon, a fundamental process in peptide chain formation. The anticodon, which interprets the mRNA information, resides at the 34th to 36th positions of the tRNA molecule. Among the three regions interacting with the codon and anticodon, the 35th and 36th positions form precise pairs with mRNA, while the 34th position forms non-complementary, irregular pairs known as “wobble pairing.”

The enzymatic modification of tRNA plays a pivotal role in ensuring accurate codon-anticodon pairing. These tRNA modifications include methylation (m), A-to-I editing at position 34, 5-carboxymethyluridine, pseudouridylation (Ψ), thiolation (s^2^U), wybutosine (yW), N6-isopentenyladenosine (i^6^A), N6-threonylcarbamoyladenosine (t^6^A), and adenine deamination in eukaryotes (2). These modifications can occur within the anticodon region or other areas of the molecule. Bacterial tRNA molecules commonly feature an average of about 8 modifications per molecule, while eukaryotic tRNAs typically present approximately 13 modifications per molecule (3). Any dysfunction or mutations in genes that regulate these nuanced adjustments can instigate frameshift errors and potentially trigger intracellular abnormalities. Numerous studies have demonstrated that tRNA modifications and the enzymes catalyzing these modifications play crucial roles in various human pathologies. Diseases linked to tRNA modifications encompass neurological disorders, cardiac ailments, respiratory conditions, cancer, metabolic disorders, and diseases related to mitochondrial dysfunction (4).

A key modification at anticodon sites is the t^6^A modification at the 37th position, which interprets “ANN” codons and results in a broad range of effects in both prokaryotes and eukaryotes (5, 6). The biosynthesis of t^6^A involves a two-step enzymatic process: the generation of threonylcarbamoyl-adenylate (TC-AMP) and the subsequent transfer of the TC component to tRNA. In bacteria, the formation of the TC-AMP intermediate is facilitated by enzymes TsaC or TsaC2 and involves bicarbonate, threonine, and ATP. The TC moiety is then attached to position A37 of the tRNA by the TsaB-TsaD-TsaE complex (7). In eukaryotes, Sua5 (analogous to bacterial TsaC) catalyzes the biosynthesis of TC-AMP. The loss of Sua5 leads to imprecise scanning of the AUG codon and a higher frequency of both +1 frameshift and nonsense suppression events (8). In the eukaryotic context, either the KEOPS (kinase, putative endopeptidase, and other proteins of small size) complex or Qri7 mediates the transfer of the TC moiety from TC-AMP to tRNA (9, 10). Importantly, the KEOPS complex exists in both the cytosol and the nucleus and consists of a linearly organized set of multiple subunits, such as Gon7, Pcc1, Kae1, Bud32, and Cgi121 in *Saccharomyces cerevisiae*. On the other hand, Qri7 functions as an independent unit in the mitochondria and serves as a structural and functional paralog of Kae1 (9).

In addition to their well-established role in t^6^A tRNA modification, the KEOPS complex and Qri7 have been implicated in regulating a range of cellular processes in eukaryotic organisms. For instance, in *S. cerevisiae*, the KEOPS complex is involved not only in t^6^A tRNA modification but also in telomere replication and transcriptional regulation (11, 12). Qri7 contributes to mitochondrial genome stability and morphology in *S. cerevisiae* (13). In *Caenorhabditis elegans*, OSGEPL1, a Qri7 ortholog, also plays a similar role in mitochondrial genome stability (13).

Recently, we conducted an in-depth analysis of the various biological roles of the KEOPS complex in *Cryptococcus neoformans* (Cn), a fungal pathogen notorious for causing meningoencephalitis worldwide (14). Structurally, the CnKEOPS complex is linearly organized as Pcc1-Kae1-Bud32-Cgi121 but notably lacks a Gon7-like ortholog found in *S. cerevisiae*. When any component of the CnKEOPS complex is deleted, we observed significant abnormalities in multiple aspects of the fungal biology, including vegetative growth, cell cycle control, sexual differentiation, production of protective structures like capsule and melanin, as well as in t^6^A tRNA modification (14). Furthermore, the CnKEOPS complex acts as a significant transcriptional regulator, influencing hundreds of genes related to carbon and nitrogen metabolism, sterol biosynthesis, mating, and other vital cellular functions. Given its wide-ranging impacts, the absence of any KEOPS component significantly diminishes the virulence of *C. neoformans* (14). However, it remains an open question whether the diverse functionalities of the CnKEOPS complex stem from its role in tRNA modification or other cellular activities.

In this study, we aimed to address two main questions. First, we wanted to determine the extent to which t^6^A tRNA modification contributes to the diverse functions of the CnKEOPS complex in *C. neoformans*. Second, we sought to explore the pathobiological roles of Qri7, a mitochondrial protein that is a functional paralog of the KEOPS complex. To tackle these questions, we performed functional analyses of two key proteins, Sua5 and Qri7, within this fungal pathogen. Our findings reveal that the wide-ranging roles of the CnKEOPS complex are largely mediated by its involvement in t^6^A modification, facilitated by Sua5. Interestingly, we found that Qri7 appears to be mostly inconsequential in the context of *C. neoformans*. This study serves as the first comprehensive exploration of the roles of Sua5 and Qri7 in fungal pathogens, thereby enriching our understanding of the significance of t^6^A tRNA modification in fungi.

## RESULTS

### Identification of Sua5 and its roles in the t^6^A tRNA modification of *C. neoformans*

**Fig 1:**
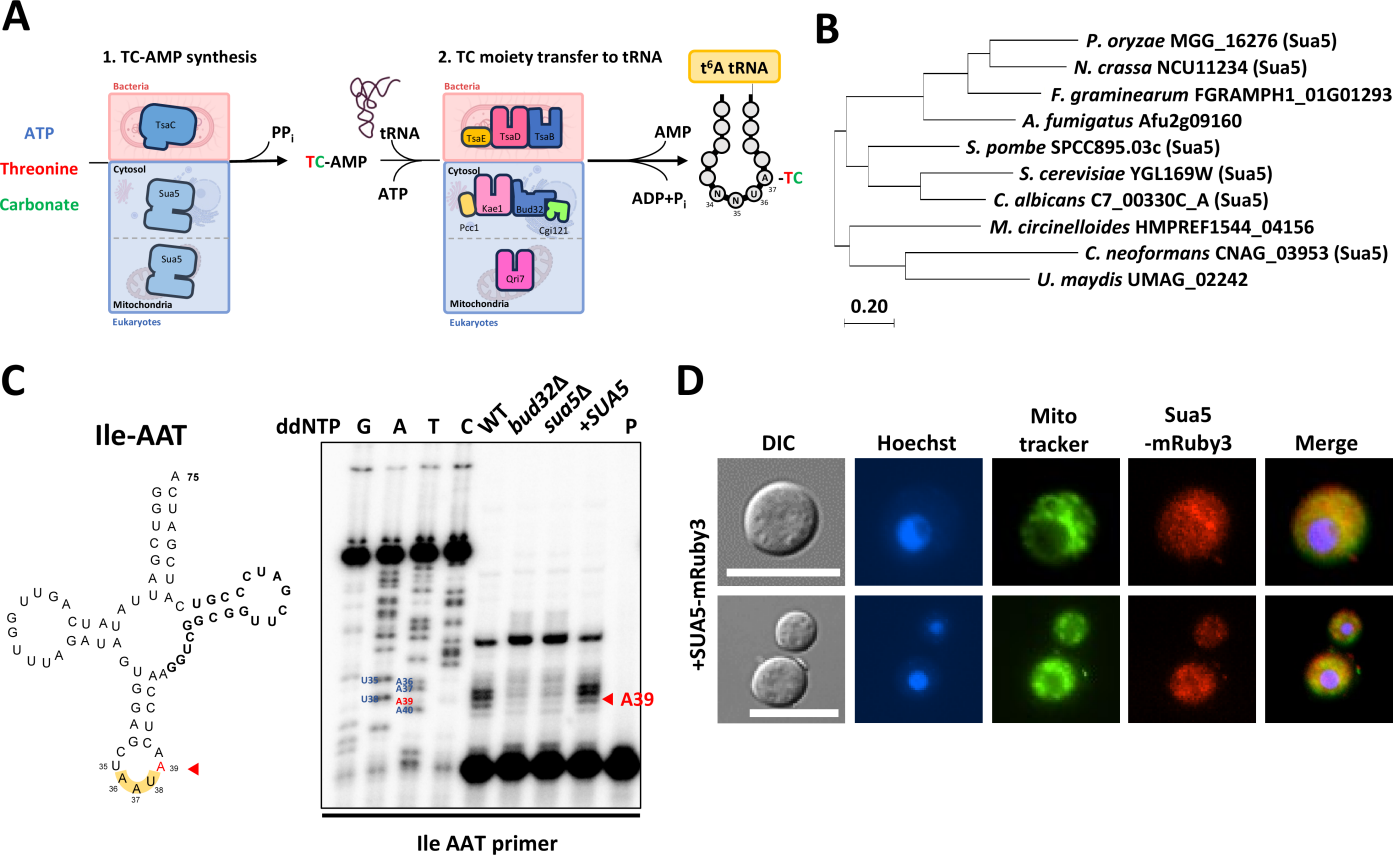
The role of Sua5 in t^6^A tRNA modification in *C. neoformans*. (**A**) Schematic representation of t^6^A tRNA modification pathways in bacteria and eukaryotes. The precursor threonylcarbamoyl-adenylate (TC-AMP) is synthesized by bacterial TsaC and eukaryotic Sua5, utilizing ATP, threonine, and carbonate. In bacteria, the TC moiety is transferred from TC-AMP to position A37 on the tRNA substrate by the TsaBDE complex, whereas in eukaryotes, this task is performed by the cytosolic KEOPS complex and mitochondria protein Qri7. (**B**) Phylogenetic analysis of fungal Sua5 orthologs. Protein sequences were sourced from FungiDB (https://fungidb.org). Evolutionary analyses were performed using MEGA11. (**C**) Primer extension analysis of tRNA Ile (AAU). Total RNAs isolated from wild-type (WT, H99S), *bud32*Δ (YSB1980), *sua5*Δ (YSB10685), and *sua5*Δ::*SUA5-mRuby3* (+*SUA5*, YSB10690) strains were hybridized with a 5′-end-labeled primer (Cn_Ile-AAT-R) for extension. A PCR product of tRNA Ile (AAU) served as a sequencing ladder template. The t^6^A-modified base (A39) is indicated by a red arrow both in the cloverleaf structure of Ile tRNA and the primer extension data. The “P” lane contains only the primer. (**D**) Subcellular localization of Sua5. Cells expressing mRuby3-tagged Sua5 (*sua5*Δ::*SUA5-mRuby3*; YSB10690) were fixed and stained with Hoechst dye for nuclear visualization and Mitotracker dye to highlight the mitochondria. Scale bar = 10 µm.

Sua5 is a universally conserved protein that initiates the process of t^6^A RNA modification (Fig. 1A). In the yeast species *S. cerevisiae*, Sua5 is distributed throughout the cell, including in both the cytosol and the mitochondria, and serves multiple functions (11). To identify the Sua5 ortholog in *C. neoformans*, we conducted a BLAST search using *S. cerevisiae* Sua5 (YGL169W) protein sequence in the *C. neoformans* H99 strain genome database of the fungiDB (https://fungidb.org/fungidb/app). This led us to identify the *C. neoformans* Sua5 ortholog, designated as CNAG_03953 (Fig. 1B).

To explore the specific functions of Sua5 in t^6^A tRNA modification within *C. neoformans*, we generated deletion mutants of the *SUA5* gene using the *C. neoformans* H99S strain as a background (Fig. S1A). We then examined whether Sua5 plays an expected role in t^6^A tRNA modification via its involvement in TC-AMP biosynthesis. Building upon our previous work, which highlighted the role of the KEOPS complex in t^6^A tRNA modification (14), we conducted a primer extension analysis using t^6^A-containing tRNA (Ile AAU) extracted from wild-type, *bud32*Δ, and *sua5*Δ strains. The primer extension profiles revealed that the *sua5*Δ mutant closely resembled the *bud32*Δ but markedly diverged from the wild-type strain at nucleotide position 39, corresponding to position 37 in the *S. cerevisiae* tRNA (Ile AAU) (Fig. 1C).

To corroborate our findings, we generated *sua5*Δ::*SUA5-mRuby3* complemented strains. In these strains, the *mRuby3* allele was in-frame fused to the C-terminus of *SUA5* and reintegrated into the native locus of *SUA5* in the *sua5*Δ mutant (Fig. S1B). The cellular distribution pattern of Sua5 was pervasive throughout the cell rather than concentrated in specific organelles (Fig. 1D), mirroring that of its *S. cerevisiae* counterpart, which is known to localize in the cytoplasm, nucleus, and mitochondria (15). This uniform distribution is also consistent with earlier observations regarding the *C. neoformans* KEOPS complex (14). Importantly, reintroducing the *SUA5-mRuby3* allele into the *sua5*Δ mutant successfully restored the wild-type primer extension pattern at position 39 of the Ile tRNA (Fig. 1C). Overall, these results confirm that Sua5 plays a conserved and pivotal role in the t^6^A tRNA modification in *C. neoformans*.

### Roles of t^6^A tRNA modification in the growth and differentiation of *C. neoformans*

**Fig 2:**
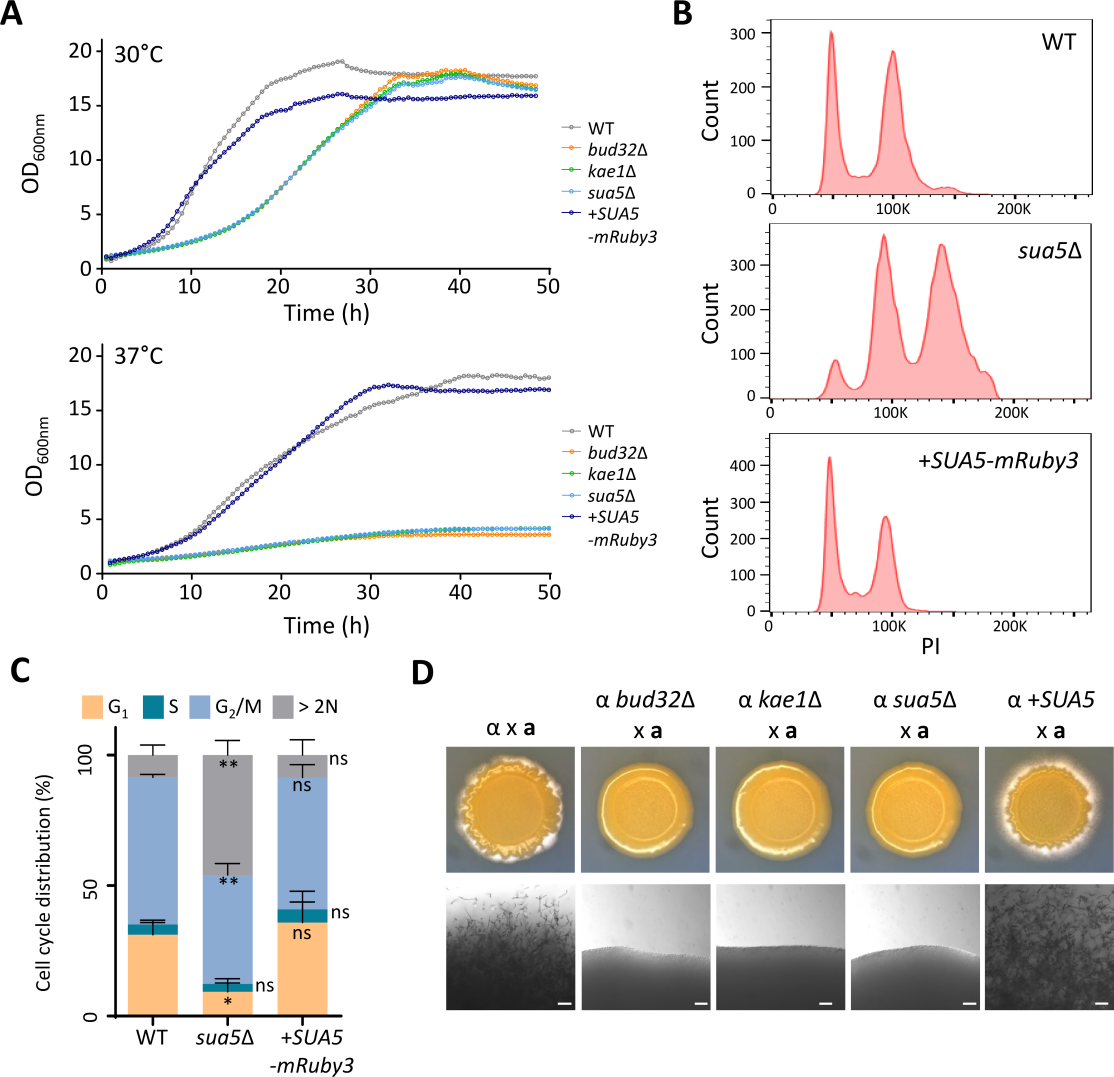
Roles of Sua5 in the growth and differentiation of *C. neoformans*. (**A**) Quantitative growth rates for wild-type (WT; H99S) and various mutant strains [*bud32*Δ (YSB1968), *kae1*Δ (YSB4863), *sua5*Δ (YSB10685), and *sua5*Δ::*SUA5-mRuby3* (YSB10690)] were assessed at 30°C and 37°C using a multi-channel bioreactor. (**B**) Flow cytometry analysis was used to evaluate cell cycle phases in cells stained with propidium iodide (PI). The flow cytometry graphs show representative data of three biological replicates (Fig. S2). (**C**) Cell populations in the flow cytometry analysis were segregated into G1, S, and G2/M phases as shown in Fig. S2. The proportion of cell populations from three biological replicates was quantified. One-way ANOVA was employed to detect statistically significant differences between the WT and mutants. Error bars represent the standard error of the mean (SEM). Statistical significance levels are denoted as follows: ns (not significant), * (0.01 < *P* < 0.05), ** (0.001 < *P* < 0.01). (**D**) Filamentation and mating efficiency were evaluated for the Sua5 mutant. *MAT*α and *MAT*a strains were co-cultured on V8 medium (pH 5.0) for 10 days at room temperature in the dark. Strains include α (H99) × **a** (KN99a), α *bud32*Δ (YSB1968) × **a**, α *kae1*Δ (YSB4863) × **a**, α *sua5*Δ (YSB10685) × **a**, and α *sua5*Δ::*SUA5-mRuby3* (+*SUA5*, YSB10690) × **a**. Scale bar = 10 µm.

While Sua5 is not a component of the KEOPS complex, it plays a crucial role in t^6^A tRNA modification, as shown in Fig. 1C. To dissect the functional contributions of this modification to the diverse pathobiological roles of the KEOPS complex, we conducted comparative phenotypic analyses using *sua5*Δ, *kae1*Δ, and *bud32*Δ mutants. Initial assessments focused on the role of Sau5 in growth and cell cycle regulation in *C. neoformans*. Relative to the wild-type strain, the *sua5*Δ mutant displayed severe growth defects at 30°C, comparable to those observed in *kae1*Δ and *bud32*Δ mutants (Fig. 2A). These growth defects became even more pronounced at the host physiological temperature (37°C) (Fig. 2A). Complementation with the *SUA5-mRuby* allele effectively restored normal growth in the *sua5*Δ mutant. Furthering our investigation, fluorescence-activated cell sorting (FACS) analysis revealed significant cell cycle irregularities in the *sua5*Δ mutant, including a notable decrease in the G1 phase duration and an unusual peak beyond the 2N DNA content (Fig. 2B and C; Fig. S2). These abnormalities align with previously documented cell cycle defects in KEOPS complex mutants, such as *pcc1*Δ, *kae1*Δ, *bud32*Δ, and *cgi121*Δ (14).

The KEOPS complex has been shown to affect the sexual differentiation of *C. neoformans* (14). Deletions of *PCC1*, *KAE1*, *BUD32*, or *CGI121* all result in a substantial reduction in the unilateral mating paired with the *MAT***a** wild-type strain (KN99**a**) (14). To evaluate the role of Sua5 in sexual differentiation, we mated the *MAT*α *sua5*Δ mutant with the *MAT***a** wild-type strain. In line with previous observations, the *sua5*Δ mutant exhibited severely reduced filamentous growth, akin to the *bud32*Δ and *kae1*Δ mutants (Fig. 2D). Importantly, the *sua5*Δ::*SUA5-mRuby3* (+*SUA5*) complemented strain exhibited mating capabilities comparable to the wild-type (Fig. 2D), confirming Sua5’s role in this biological process. Collectively, our findings strongly indicate that t^6^A tRNA modification is pivotal for both the growth and differentiation of *C. neoformans*.

### Roles of t^6^A tRNA modification in stress responses and virulence factor production

**Fig 3:**
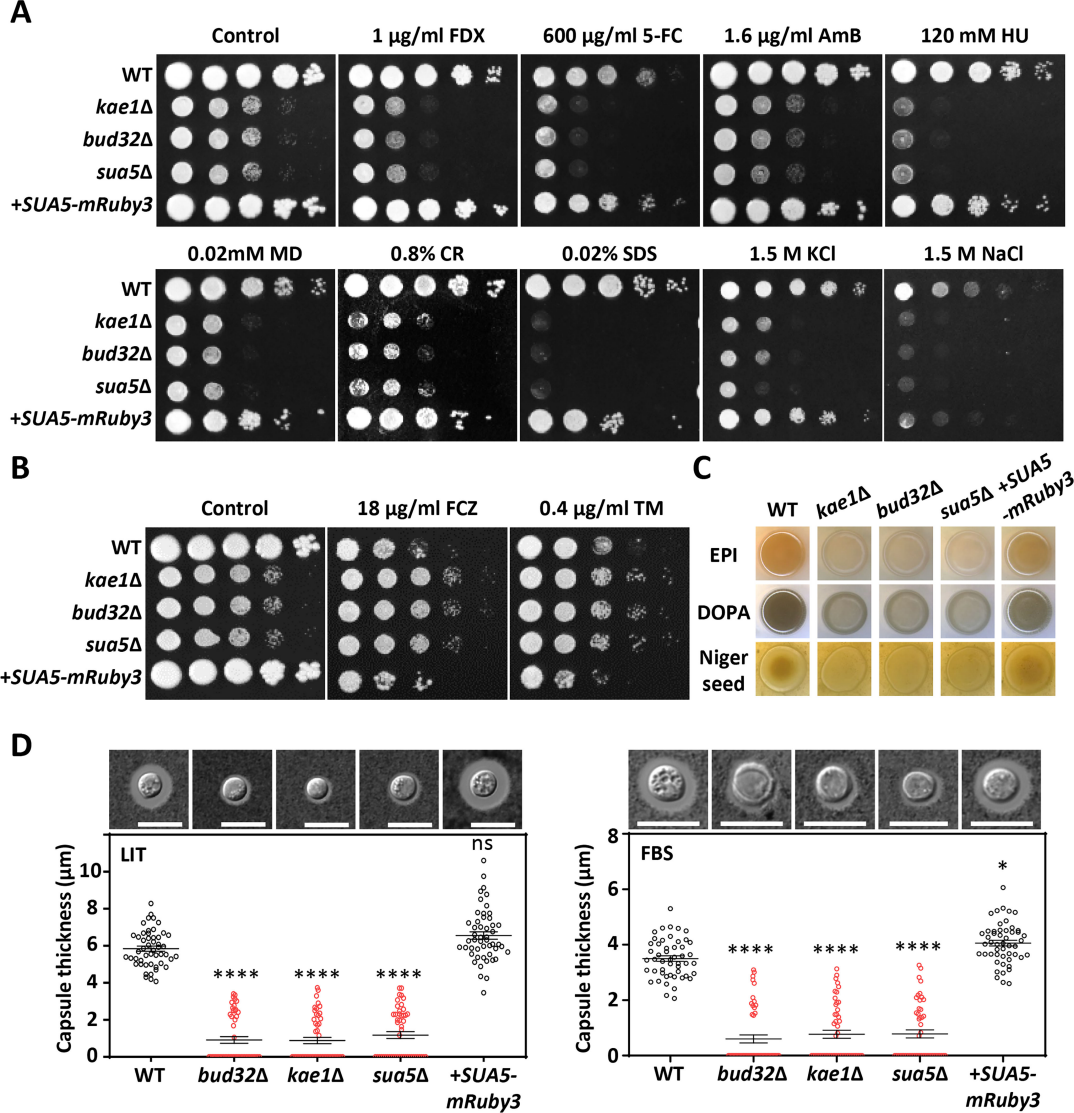
Roles of Sua5 in stress responses and virulence factor production in *C. neoformans*. (**A and B**) Susceptibility to antifungal agents and stress inducers was evaluated in wild-type (H99S) *kae1*Δ (YSB4863), *bud32*Δ (YSB1968), *sua5*Δ (YSB10685), and *sua5*Δ::*SUA5-mRuby3* (YSB10690) strains. Strains were cultured overnight in YPD medium at 30°C, serially diluted (1 to 10^4^), and spotted onto YPD plates containing the indicated concentration of antifungal agents or stress inducers: fludioxonil (FDX), 5-fluorocytosine (5-FC), amphotericin B (AmB), hydroxyurea (HU), menadione (MD), Congo red (CR), sodium dodecyl sulfate (SDS), KCl, NaCl, fluconazole (FCZ), or tunicamycin (TM). Plates were incubated at 30°C for 3–4 days. (**C**) Melanin production was tested by spotting overnight cultured strains onto Niger seed, L-DOPA, and epinephrine agar medium containing 0.1% glucose, followed by incubation at 37°C and documentation after 1–3 days. (**D**) Capsule production was assessed by culturing each strain in capsule-inducing media [Littman (LIT) or fetal bovine serum (FBS)] at 37°C for 2 days. Capsule thickness was quantified through India ink staining in 50 cells per strain. Statistical significance was determined using one-way ANOVA with Bonferroni’s multiple comparison test. Data are shown as mean values with the standard error of the mean (SEM). Statistical significance levels are denoted as follows: ns (not significant), * (0.01 < *P* < 0.05), **** (*P* < 0.0001).

Extending our investigation, we examined whether the *sua5*Δ mutant shares similar pathobiological characteristics with the KEOPS complex mutants, specifically *kae1*Δ and *bud32*Δ. As previously described (14), both *kae1*Δ and *bud32*Δ mutants exhibit heightened susceptibility to a range of antifungal agents and stressors, including antifungal drugs (fludioxonil, 5-fluorocytosine, and amphotericin B), genotoxic agents (hydroxyurea), a specific oxidative stress agent (menadione), cell membrane stress (SDS), cell wall stress (Congo red), and osmotic stress (KCl and NaCl) (Fig. 3A). In contrast, these mutants showed increased resistance to fluconazole and ER stress inducer, tunicamycin (Fig. 3B). Notably, the *sua5*Δ mutant mirrored these responses to stress agents (Fig. 3A and B).

In the context of virulence, the KEOPS complex mutants are markedly compromised in the production of two major virulence factors in *C. neoformans*: melanin and capsule (14). Melanin, a polyphenolic pigment, anchors to the chitin layer of the cryptococcal cell surface and serves as both an antioxidant and an antiphagocytic factor (16). Polysaccharide capsules, which form the outermost layer of the cryptococcal cell surface, act as another primary antiphagocytic factor (17). Consistent with the profiles of *kae1*Δ and *bud32*Δ mutants, the *sua5*Δ mutant displayed a significant reduction in the production of melanin (Fig. 3C) and capsule (Fig. 3D). Taken together, our findings strongly suggest that the versatile functions of the KEOPS complex in stress responses and virulence factor production are fundamentally linked to its role in t^6^A tRNA modification. Specifically, Sua5, which supplies the TC-AMP substrate for the KEOPS complex, exhibits identical functional roles, underlining the integral nature of t^6^A tRNA modification in these processes.

### The dispensable role of Sua5 in the mitochondria functions in *C. neoformans*

**Fig 4:**
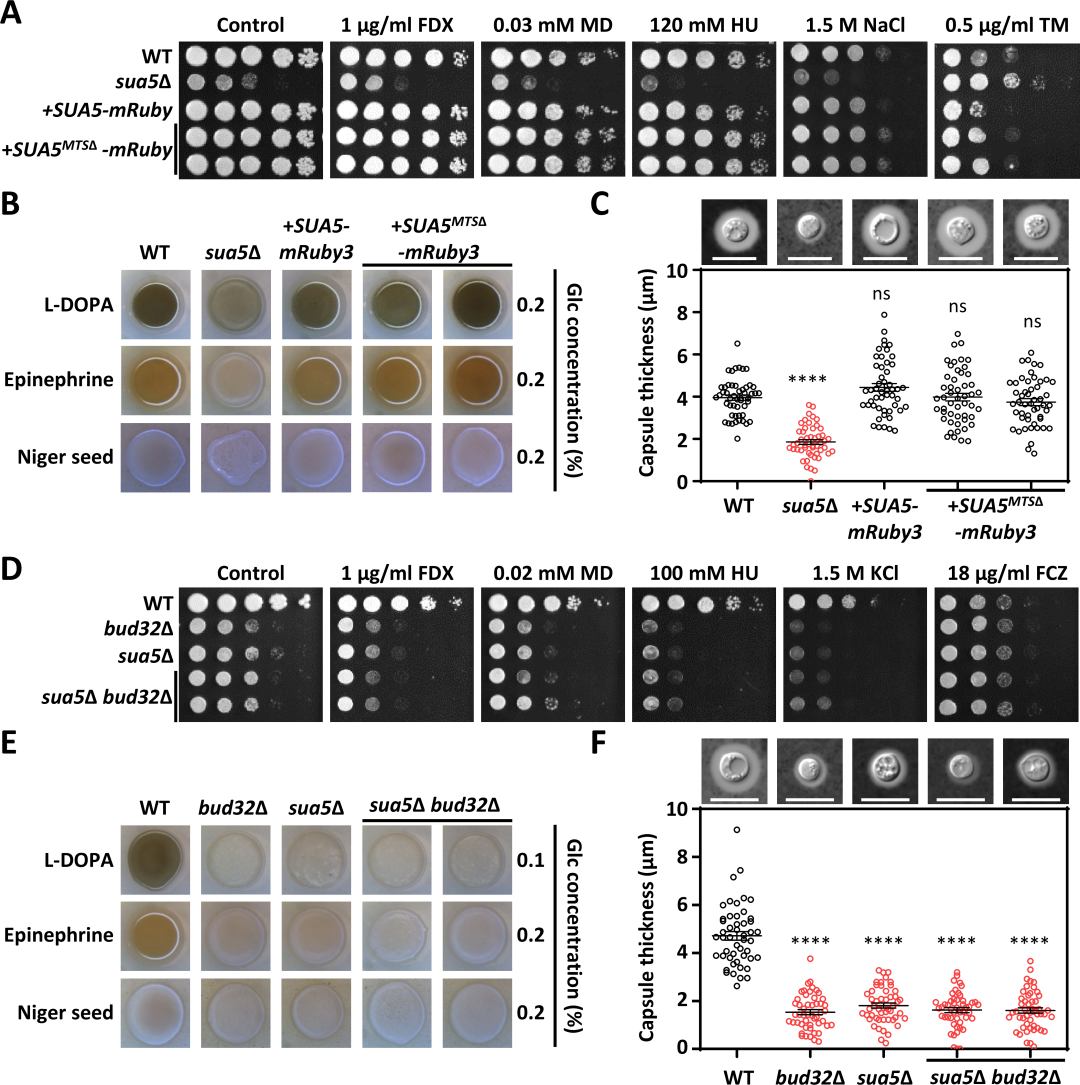
Roles of the mitochondrial Sua5 in *C. neoformans*. (**A, D**) Susceptibility to antifungal agents and stress inducers was evaluated in wild-type (H99S), *sua5*Δ (YSB10685), *sua5*Δ:: *SUA5-mRuby3* (YSB10690), *sua5*Δ:: *SUA5^MTS^*^Δ^-*mRuby3* (YSB11178 and YSB11179), *bud32*Δ (YSB1968), and *sua5*Δ *bud32*Δ (YSB11182 and YSB11183) strains. Strains were cultured overnight in YPD broth at 30°C, serially diluted (1 to 10^4^), and spotted onto YPD plates containing the indicated concentration of fludioxonil (FDX), menadione (MD), hydroxyurea (HU), NaCl, KCl, tunicamycin (TM), or fluconazole (FCZ). (**B, E**) Melanin production was tested by spotting overnight cultured strains onto Niger seed, L-DOPA, and epinephrine agar medium containing 0.2% glucose, followed by incubation at 37°C and documentation after 2–3 days. (**C, F**) Capsule production was assessed by culturing each strain in Littman’s medium (LIT) at 37°C for 2 days. Statistical significance was determined using one-way ANOVA with Bonferroni’s multiple comparison test. Data are shown as mean values with the standard error of the mean (SEM). Statistical significance levels are denoted as follows: ns (not significant), **** (*P* < 0.0001).

In *S. cerevisiae*, the t^6^A tRNA modification process occurs both in the cytosol and mitochondria, facilitated by its association with the KEOPS complex and Qri7, respectively. Both pathways are supplied with the TC-AMP substrate by Sua5 (10). However, our observations indicate that *sua5*Δ mutants in *C. neoformans* exhibit phenotypes indistinguishable from those of KEOPS mutants, suggesting that the mitochondrial role of Sua5 may be non-essential in this organism. To test this hypothesis, we engineered a mutant with an *SUA5* allele devoid of the mitochondrial targeting sequence (MTS). Computational analysis using MitoFates and Target P revealed a 22-amino acid MTS at the N-terminus of the cryptococcal Sua5 (Fig. S4A). We generated a *SUA5^MTS^*^Δ^*-mRuby3* allele and reintegrated it into the native *SUA5* locus of the *sua5*Δ mutant (Fig. S4B). Intriguingly, the resultant *sua5*Δ:: *SUA5^MTS^*^Δ^-*mRuby3* strain exhibited a stress response indistinguishable from both the wild-type and the *sua5*Δ::*SUA5-mRuby3* strains (Fig. 4A). This indicates that Sua5’s mitochondrial localization is not crucial for its cellular functions in *C. neoformans*. Additionally, the *sua5*Δ:: *SUA5^MTS^*^Δ^-*mRuby3* strain exhibited capsule and melanin production at levels comparable to those of the wild-type (Fig. 4B and C). To further substantiate this claim, we generated *sua5*Δ *bud32*Δ double mutants (Fig. S5) and assessed their stress response phenotypes to their corresponding single mutants (Fig. 4D). In line with the findings shown in Fig. 4A, the *sua5*Δ *bud32*Δ double mutant did not exhibit increased susceptibility to stress or differences in capsule and melanin production when compared to the *sua5*Δ or *bud32*Δ single mutants (Fig. 4D through F). These findings lead us to conclude that, unlike in *S. cerevisiae* where Sua5 plays a dual role in tRNA modification, in *C. neoformans*, Sua5’s primary function appears to be in coordination with the KEOPS complex. Thus, mitochondrial tRNA modification may not be a crucial aspect of its role. Consequently, we predict that the multifunctional attributes of the KEOPS complex are likely facilitated through its collaboration with Sua5.

### Roles of Qri7 in mitochondrial functions in *C. neoformans*

**Fig 5:**
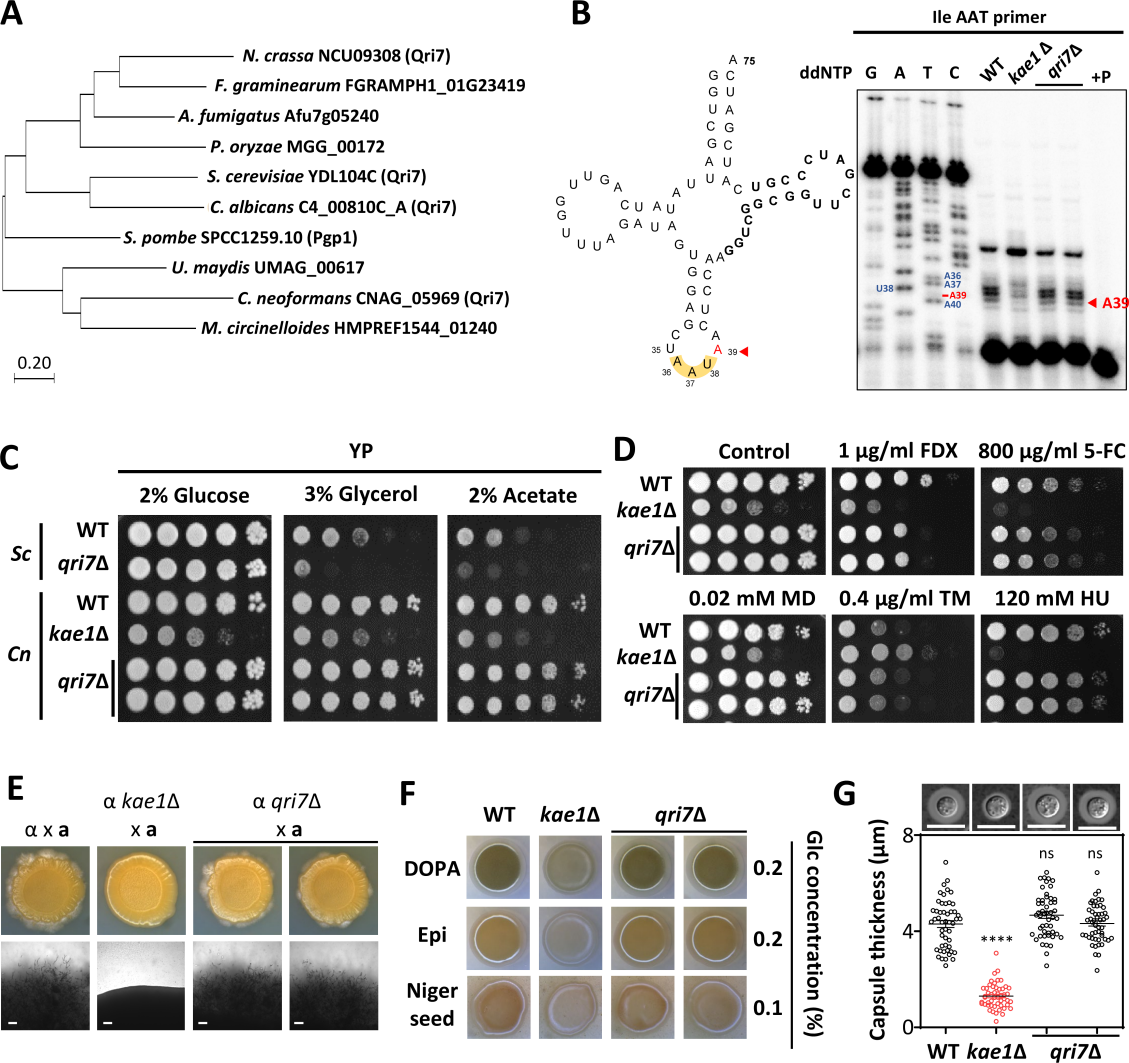
Roles of Qri7 in *C. neoformans*. (**A**) Phylogenetic tree of fungal Qri7 orthologs. Protein sequences were sourced from FungiDB (https://fungidb.org). Evolutionary analyses were conducted using MEGA11. (**B**) The role of Qri7 in t^6^A tRNA modification. Primer extension analysis of tRNA Ile (AAU) was performed with total RNAs from wild-type (H99S), *kae1*Δ (YSB4863), and *qri7*Δ (YSB10692 and YSB10693) strain. Each RNA sample was hybridized with a 5′-end-labeled primer (Cn_Ile-AAT-R) for extension. A PCR product of tRNA Ile (AAU) served as a sequencing ladder template. The t^6^A-modified base (A39) is indicated by a red arrow both in the cloverleaf structure of Ile tRNA and the primer extension data. The “P” lane contains only the primer. (**C**) The role of Qri7 in the growth of non-fermentable carbon sources. *S. cerevisiae* (*Sc*) wild-type (BY4742), *Sc qri7*Δ (138F-3), *C. neoformans* (*Cn*) wild-type (H99S), *Cn kae1*Δ (YSB4863), and *Cn qri7*Δ (YSB10692 and YSB10693) strains were cultured in YPD broth at 30°C, serially diluted (1 to 10^4^), and spotted onto YP based containing different carbon sources (2% glucose, 3% glycerol, or 2% acetate). The plates were cultured at 30°C for 4 days. (**D**) Each strain was cultured, serially diluted, and spotted as described in Fig. 3A and B onto YPD medium containing FDX, AmB, 5-FC, MD, TM, and HU, and then incubated at 30°C for 4 days. (**E**) Mating assay. The following *MAT*α and *MAT*a strains were co-cultured on V8 medium (pH 5.0) for 10 days at room temperature in the dark: α (H99) × **a** (KN99a), α *kae1*Δ (YSB4863) × **a**, and α *qri7*Δ (YSB10692 and YSB10693) × **a**. (**F**) Melanin production was tested by spotting overnight cultured strains onto Niger seed, L-DOPA, and epinephrine agar medium containing 0.1% or 0.2% glucose, followed by incubation at 37°C and documentation after 1–3 days. (**G**) Capsule production was assessed by culturing each strain in Littman’s medium (LIT) at 37°C for 2 days. Statistical significance was determined using one-way ANOVA with Bonferroni’s multiple comparison test. Data are shown as mean values with the standard error of the mean (SEM). Statistical significance levels are denoted as follows: ns (not significant), **** (*P* < 0.0001).

In *S. cerevisiae*, Qri7 serves as a Kae1 paralog and plays a significant role in mitochondria t^6^A tRNA modification (10). To further corroborate the notion that mitochondria t^6^A tRNA modification is not essential in *C. neoformans*, we set out to investigate the functions of its Qri7 ortholog. Using the *S. cerevisiae* Qri7 protein sequence for a BLAST search in FungiDB, we identified a single orthologous *QRI7* gene (CNAG_05969) in *C. neoformans* (Fig. 5A). We then generated two independent *qri7*Δ mutants and assessed their phenotypes in relation to the *sua5*Δ and *kae1*Δ mutants (Fig. S6). To evaluate the effect of *QRI7* deletion on intracellular tRNA modifications, we performed primer extension assays on whole-cell RNA and compared the results with those from the *kae1*Δ mutant. Our analysis confirmed that the *qri7*Δ mutation does not have a significant impact on overall tRNA modification. However, as we were unable to obtain sufficient mitochondrial tRNA within the cells, we could not specifically assess the role of Qri7 in mitochondrial t^6^A tRNA modifications.

In *S. cerevisiae*, the deletion of *QRI7* leads to growth impairments when non-fermentable carbon sources are used (18) (Fig. 5C). However, we found that the deletion of *QRI7* in *C. neoformans* had no significant impact on growth when cultured with 3% glycerol or 2% acetate (Fig. 5C). When we examined the growth patterns of strains *sua5*Δ, *sua5*Δ:: *SUA5^MTS^*^Δ^-*mRuby3*, and *bud32*Δ *sua5*Δ in the presence of a fermentable carbon source (2% glucose) or non-fermentable carbon sources (3% glycerol or 2% acetate), we observed no discernible growth variations in the *SUA5* mutants (Fig. S7). Given that Sua5 is the sole enzyme responsible for initiating t^6^A tRNA modification across the cytoplasm and mitochondria, our results suggest that t^6^A tRNA modification is not crucial for growth on non-fermentable carbon sources in *C. neoformans*.

To investigate the potential roles of Qri7 in *C. neoformans*, we assessed the capacity of *qri7*Δ mutants to respond to various stress conditions, undergo sexual differentiation, and produce two major virulence factors, melanin and capsule. Unlike the KEOPS complex mutants, the *qri7*Δ mutant did not exhibit any noticeable changes in growth, stress responses, antifungal drug resistance, differentiation, and virulence factor production (Fig. 5D through G). These collective findings support the hypothesis that Qri7, mitochondrial Kae1 ortholog, is not crucial for the pathobiological functions of *C. neoformans*.

## DISCUSSION

The t^6^A tRNA modification is a highly conserved and critical factor for maintaining translation fidelity across all living organisms. Despite its universal importance, its specific role in fungal pathogens remains largely unexplored. Our previous research established the critical role of the KEOPS complex, organized in a Pcc1-Kae1-Bud32-Cgi121 linear arrangement, in facilitating t^6^A tRNA modification in the opportunistic human fungal pathogen *C. neoformans*. However, it was ambiguous whether the KEOPS complex’s diverse functions in pathobiological processes were directly mediated by its role in tRNA modification. In the current study, we focused on the protein Sua5, which is specifically involved in t^6^A tRNA modification by catalyzing the initial step of TC-AMP production in *C. neoformans*. Our findings demonstrate that the diverse biological roles of the KEOPS complex are indeed dependent on Sua5. Additionally, we discovered that Qri7, a mitochondrial Kae1 ortholog, has minimal impact on cellular growth, stress response, and virulence factor formation in *C. neoformans*. This contrasts sharply with the essential roles played by the cytosolic KEOPS complex.

The KEOPS complex is a critical regulator of cytosolic t^6^A tRNA modifications in a broad range of organisms, underscoring its highly conserved nature (19). While the two-step process of precursor formation and its subsequent transfer onto tRNA are conserved across species (19), variations exist in the number and functions of subunits that execute the second step. Notably, Sua5, responsible for catalyzing the initial step, is universally conserved as a single unit across all known organisms (19). This highlights its pivotal role in tRNA modification, a significance likely rooted in its evolutionary conservation. This notion is further supported by previous and current studies in *S. cerevisiae* and *C. neoformans*, where the deletion of *SUA5* leads to a noticeable growth defect in both organisms (20).

The pronounced growth defect observed in the *sua5*Δ mutant underscores the vital pathobiological role of Sua5. Our previous research showed that the *bud32*∆ mutant, a member of the KEOPS mutants, is avirulent in both a murine model of systemic cryptococcosis and an insect model (14). Given that the *sua5*∆ mutant mirrors the KEOPS mutants in its marked growth defects at the host’s physiological temperature of 37°C, increased sensitivity to various external stresses, and notable deficiencies in the formation of crucial virulence factors such as capsule and melanin, we expected *sua5*∆ to exhibit diminished or no virulence in *C. neoformans*.

In *S. cerevisiae*, Sua5 and the KEOPS complex work in tandem to regulate tRNA modification in the cytosol and telomere maintenance in the nucleus (11). However, our prior research in *C. neoformans* indicated that the KEOPS complex is involved in t^6^A tRNA modifications but not in regulating telomere length (14). The present study builds upon this, revealing that in *C. neoformans*, Sua5 is also implicated in t^6^A tRNA modifications, and its function aligns closely with that of the KEOPS complex. Given this, we hypothesize that Sua5 is unlikely to be involved in telomere maintenance in this particular fungal pathogen. Nonetheless, additional roles that Sua5 may play in *C. neoformans* remain to be further investigated in future studies.

The domain of Sua5 in *C. neoformans* consists of a TsaC-like domain and an additional Sua5 domain, and this characteristic is observed in all fungi, including *S. cerevisiae*. However, in other eukaryotes and bacteria, there is no clear phyletic pattern showing either the presence of TsaC alone or the additional Sua5 domain (21). However, the functional significance of this additional Sua5 domain in fungi species remains enigmatic. Given this context, it is conceivable that the extra domain found in fungal Sua5 orthologs may facilitate a broader range of functions through interactions with multiple substrates. This hypothesis warrants further exploration in future research endeavors.

In *S. cerevisiae*, Sua5 is known to localize not only in the cytosol and nucleus but also in the mitochondria, where it may independently function alongside Qri7, a mitochondrial Kae1 paralog (15). Similarly, our findings confirm the presence of Sua5 in the mitochondria of *C. neoformans*. However, our study presents a compelling body of evidence suggesting that the mitochondrial function of Sua5 is largely inconsequential in *C. neoformans*. Several key observations support this claim. First, the *sua5*Δ mutant exhibited similar phenotypes to the *bud32*Δ and *kae1*Δ mutants. Second, re-introducing the *SUA5* allele deleted of a mitochondrial targeting sequence (*SUA5^MTS^*^Δ^) into the *sua5*Δ mutant fully restored wild-type characteristics. Third, the deletion of *QRI7* did not result in any observable phenotypic changes. Finally, the *sua5*Δ *bud32*Δ double mutant displayed phenotypes identical to those of the *bud32*Δ mutant alone. This series of observations further underscores the organism-specific intricacies of tRNA modification pathways.

The precise function of Qri7 in *C. neoformans* remains enigmatic. Our bioinformatics analysis indicates that Qri7 is highly conserved across various fungal species, indicating its potential functional significance. Interestingly, *S. cerevisiae* Qri7 can substitute for TsaBDE, the bacterial ortholog of the KEOPS complex, and facilitate t^6^A synthesis *in vitro* when paired with the bacterial Sua5 ortholog, TsaC (15). Similarly, both Sua5 and Qri7 are capable of t^6^A synthesis *in vitro* in the yeast model (15). Moreover, overexpression of a mitochondria-targeting sequence-deleted *QRI7* allele (*QRI7^MTS^*^Δ^) can ameliorate t^6^A tRNA modification deficiencies in *kae1*Δ mutants, suggesting a possible functional overlap between Qri7 and its cytosolic paralog, Kae1 (11). However, the specific role of Qri7 in mitochondrial t^6^A tRNA modification remains unclear. Future studies will be necessary to determine whether the *QRI7^MTS^*^Δ^ allele can substitute for the function of Kae1 in *C. neoformans*. We conducted a BLAST search in *C. neoformans* to identify potential paralogs of Kae1 and Qri7, but found none. Both Kae1 and Qri7 feature the tRNA N6-adenosine threonylcarbamoyltransferase domain, a signature absent in other cryptococcal proteins. The third candidate, CNAG_05260, has a high *E*-value (0.063) and lacks common domains, suggesting that the presence of Kae1 and Qri7 paralogs is improbable. Nevertheless, there remains the possibility of an unrecognized protein, distinct from Kae1 and Qri7, contributing to mitochondrial t^6^A tRNA modification in *C. neoformans*. Alternatively, other tRNA modifications might have a more central role in the mitochondria of this pathogen.

In *S. cerevisiae*, Qri7 has been linked to maintaining mitochondrial genome stability (13) and is essential for growth in conditions that require non-fermentable carbon sources (18). Contrary to these findings, our data indicate that the deletion of *QRI7* in *C. neoformans* has negligible impact on growth when using similar non-fermentable carbon sources. Based on our observations with *SUA5* mutants, it seems that t^6^A tRNA modification might not be crucial for growth on non-fermentable carbon sources in *C. neoformans*. Whether Qri7 in *C. neoformans* contributes to maintaining mitochondrial genome stability is an open question. Further studies to elucidate the unique or overlapping roles of Qri7 in diverse fungal species could yield important insights into its broader biological implications.

In summary, our research furnishes pivotal evidence that underscores the central role of t^6^A tRNA modification, as mediated by Sua5, in regulating the multifaceted biological functions of the KEOPS complex in *C. neoformans*. Given the evolutionary conservation of these features across fungal species, it is likely that the contributions of t^6^A tRNA modification to fungal pathogenicity are similarly conserved among fungal pathogens. This hypothesis presents a compelling avenue for future research, particularly in understanding the role of t^6^A tRNA modification across a range of fungal pathogens affecting both plants and animals.

## MATERIALS AND METHODS

### Strains and growth conditions

The strains used in this study are listed in Table S1. YPD plates containing 2% peptone, 1% yeast extract, 2% dextrose, and 2% Bacto agar were used for *C. neoformans* yeast cells at 30°C. Nourseothricin (100 µg/mL), G418 (50 µg/mL), or hygromycin B (150 µg/mL) were added to the YPD medium to select *C. neoformans* transformants constructed by the biolistic particle delivery system.

### Construction of mutant strains

*C. neoformans* serotype A H99S (*MAT*α) strain was used to create knockout mutants using the split marker/double-joint (DJ) PCR techniques (22, 23). Resistance markers for nourseothricin (*NAT*, nourseothricin acetyltransferase), neomycin/G418 (*NEO*, neomycin phosphotransferase), and hygromycin B (*HYG*, hygromycin B phosphotransferase) were integrated into gene disruption cassettes (22). The primer sequences used in this study are detailed in Table S2. In the first round of PCR, the flanking regions of target genes (*SUA5*, *QRI7*, and *BUD32*) were amplified by using genomic DNA from H99S as a template and specific primer pairs L1/L2 and R1/R2. The markers *NAT*, *NEO*, and *HYG* were amplified from their respective plasmids (pNAT, pNEO, and pHYG) using M13Fe and M13Re primers. In the second round of overlap PCR, we used the products from the first round of PCR as templates to construct gene disruption cassettes. We achieved this using primer pairs L1/SM2 and SM1/R2 for *NAT*, L1/GSL and GSR/R2 for *NEO*, and L1/HSM2 and HSM1/R2 for *HYG*. For biolistic transformation, the H99S strain was grown overnight in 50 mL of YPD broth at 30°C. Then, the cells were collected and resuspended in 5 mL of YPD. Approximately 200 µL of this suspension was spread onto YPD plates that contained 1 M sorbitol and then incubated for an additional 3 h at 30°C. Using a PDS-100 particle delivery system from Bio-Rad, 0.6 µm gold microcarrier beads (Bio-Rad) were coated with the PCR-amplified gene disruption cassettes. These coated microcarrier beads were then introduced into the cells using biochemical methods. Following the transformation, the cells were incubated for 4 h at 30°C to allow for the restoration of membrane integrity and were subsequently transferred to plates containing the appropriate antibiotics for selection.

### Construction of fluorescent protein-tagged strains

For constructing mRuby3-tagged strains, the ORF and the mitochondrial targeting signal (MTS)-deleted ORF without stop codon containing promoter regions of *SUA5* were amplified and cloned into pNEO-mRuby3ht using Gibson Assembly Master Mix Kit (New England BioLabs). The linearized plasmids pNEO_SUA5-mRuby3 and pNEO_SUA5^MTSΔ^-mRuby3, digested with SalI, were introduced into the *sua5*Δ strain through biolistic transformation. Diagnostic PCR was utilized to confirm targeted integration.

### Imaging cellular localization of mRuby3-tagged proteins

The mRuby3-tagged strains were cultured in YPD broth overnight at 30°C and subsequently subcultured in fresh YPD liquid medium until their optical density at 600_nm_ (OD_600nm_) reached 0.8. These cells were then fixed using a solution containing 4% paraformaldehyde and 3.4% sucrose for 15 min at room temperature. After fixation, the cells were centrifuged, washed with 0.1 M potassium phosphate buffer (pH 7.5) containing 1.2 M sorbitol, and then preserved in the potassium phosphate buffer. To stain the cell nuclei, the cells were exposed to 10 µg/mL of Hoechst 33342 (Thermo Fisher) in the dark for 30 min. Following this incubation, the samples were examined and imaged using both differential interference contrast (DIC) and fluorescence microscopy, specifically a Nikon Eclipse microscope equipped with a digital camera (DS-Qi2).

### Flow cytometry analysis

Flow cytometry was conducted as previously described (14). For cell preparation, the wild-type, *sua5*Δ, and *sua5*Δ::*SUA5-mRuby3* strains were cultivated until they reached an optical density at 600 nm (OD_600nm_) of 0.8. Subsequently, they were harvested and rinsed with phosphate-buffered saline (PBS). For ethanol fixation, 10^6^ cells in 300 µL of PBS were gently mixed with 700 µL of 100% ethanol and left to incubate at 4°C for 16 h. After fixation, the cells were washed with PBS containing 1% and 0.5% bovine serum albumin (BSA). The cells were then treated with 200 µg/mL of RNase A (Thermo Scientific) for 30 min at 37°C. Following centrifugation, the cells were stained with propidium iodide staining buffer [100 µg/mL propidium iodide, 100 mM Tris (pH 7.4), 150 mM NaCl, 1 mM CaCl_2_, 0.5 mM MgCl_2_, and 0.1% Nonidet P-40] for 2 h at room temperature in the dark. After a final wash with PBS and filtration through a strainer, fluorescence was measured using a BD FACS Symphony A5, recording 10,000 events per sample.

### Growth and chemical susceptibility test

Each strain was cultured in YPD broth for 16 h at 30°C, followed by a 10-fold serial dilution (1 to 10^4^). The diluted cultures were then placed onto YPD solid medium with specified concentrations of chemical agents to induce various environmental stresses. The plates were incubated at 30°C for 1–4 days, with daily photographs taken. To assess the growth rate of *SUA5* mutants, both the wild-type strain (H99S) and the mutants were incubated overnight at 30°C, and their cell concentrations were adjusted to OD_600nm_ = 0.2 in fresh YPD liquid medium. Subsequently, the cells were cultured at 30°C in a multi-channel bioreactor (Biosan Laboratories), and OD_600nm_ measurements were automatically recorded over a period of 70 h.

### Virulence factor production assay

To assess melanin production, cells cultured overnight were washed two times with PBS and then placed on agar media containing Niger seed, dopamine, or epinephrine along with 0.1% glucose. These cell-containing plates were incubated at 37°C and photographed over a period of 1–3 days. For the capsule production assay, two types of capsule-inducing media were used: fetal bovine serum (FBS) agar media (consisting of 10% fetal bovine serum and 90% PBS) and Littman’s agar media. Cells grown overnight were washed two times with PBS, and 3 µL of these cells were applied to the capsule-inducing media. The plates were then incubated at 37°C for 2 days. Subsequently, the cells were scraped, suspended in distilled water, mixed with India ink for visualization, and observed using DIC microscopy. Capsule and cell diameter measurements were performed on 50 randomly selected cells. Capsule thickness was calculated as the difference between total diameter and cell body diameter. Statistical significance was assessed using one-way ANOVA analysis, followed by Bonferroni’s multiple comparison test.

### Mating

To assess the efficiency of unilateral mating, each *MAT*α mutant and the wild-type strain (H99S) were subjected to mating with the *MAT***a** KN99**a** strain. Initially, each cell was grown in YPD broth for 16 h, then collected, and washed twice with PBS. The *MAT*α cells, at a concentration of 10^7^ cells/mL, were mixed in equal volumes with the *MAT***a** KN99**a** cells also at 10^7^ cells/mL. This cell mixture was spotted onto V8 mating media with a pH5 and incubated in the dark for a duration of 10 days. The development of filamentous growth was observed and documented using DIC microscopy (Olympus).

### Primer extension assay

Wild-type, *sua5*Δ::*SUA5-mRuby3*, and *qri7*Δ strains were cultured overnight in liquid YPD medium at 30°C. The *bud32*Δ, *sua5*Δ, and *kae1*Δ strains were cultured under the same conditions but for 48 h due to their growth defects. Cells from saturated cultures were inoculated into 25 mL of fresh YPD medium to achieve an OD_600_ of 0.2 and were grown until reaching an OD_600_ of 0.8. Subsequently, the cells were harvested and stored at −80°C until further use. The frozen cell pellet was resuspended in 1 mL of TRIzol reagent (Invitrogen) and transferred to a 2-mL screw-cap tube containing 0.3 g of acidic glass beads (Sigma). After adding 200 µL of chloroform, the mixture was homogenized using a Precellys 24 bead beater (Bertin) for 10 cycles, each consisting of 30 s of agitation at 6,500 rpm, followed by 1 min cooling at 4°C. Following centrifugation, the supernatant was mixed with 3 M sodium acetate (pH 5.3) and ice-cold isopropanol to precipitate total RNA. The RNA pellet was then washed with 80% ethanol, air-dried, and resuspended in DEPC-treated water. The primer extension assay was performed using a 5′-end γ-^32^P-ATP (Perkin Elmer)-labeled Cn_Ile-AAT-R primer (5′-ACGGGATCGAACCGCCGACC-3′). About 30 μg of total RNA was annealed with the labeled primers at 65°C for 5 min and then cooled gradually to 37°C. Primer extension was performed at 42°C for 1 h using 5 units of avian myeloblastosis virus reverse transcriptase (NEB). Sequencing ladders were generated using the DNA Cycle Sequencing Kit (Jena Bioscience) and 100 ng of the tRNA Ile (AAU) PCR product amplified from the H99 cDNA. Electrophoresis was conducted on a 15% polyacrylamide gel containing 8 M urea, and the results were visualized using a Personal Molecular Imager System (Bio-Rad).

## SUPPLEMENTAL MATERIAL

Fig. S1 msphere.00557-23-s0001.pdf Construction and verification of *SUA5* deleted and complemented strains.

Fig. S2 msphere.00557-23-s0002.pdf Monitoring of the cell cycle in the SUA5 mutant via flow cytometry.

Fig. S3 msphere.00557-23-s0003.pdf Visualization of capsule formation in *SUA5* and *KEOPS* mutants.

Fig. S4 msphere.00557-23-s0004.pdf Construction of *C. neoformans* strains lacking the mitochondria targeting sequence (MTS) in the *SUA5* allele.

Fig. S5 msphere.00557-23-s0005.pdf Construction of *sua5*Δ *bud32*Δ double mutants.

Fig. S6 msphere.00557-23-s0006.pptx Construction of *qri7*Δ mutants.

Fig. S7 msphere.00557-23-s0007.pdf The role of Sua5 in *C. neoformans* growth on non-fermentable carbon sources.

Table S1 msphere.00557-23-s0008.pdf List of strains used in this study.

Table S2 msphere.00557-23-s0009.pdf List of primers used in this study.

ASM does not own the copyrights to Supplemental Material that may be linked to, or accessed through, an article. The authors have granted ASM a non-exclusive, world-wide license to publish the Supplemental Material files. Please contact the corresponding author directly for reuse.
