# Supplementary material for: Sua5 catalyzing universal t6A tRNA modification is responsible for multifaceted functions of the KEOPS complex in Cryptococcus neoformans
Source: mSphere. 2023 Dec 12;9(1):e00557-23. doi: 10.1128/msphere.00557-23 (PMC10826353; doi:10.1128/msphere.00557-23)
Supplement: Fig. S1 — Construction and verification of SUA5 deleted and complemented strains. [file msphere.00557-23-s0001.pdf]

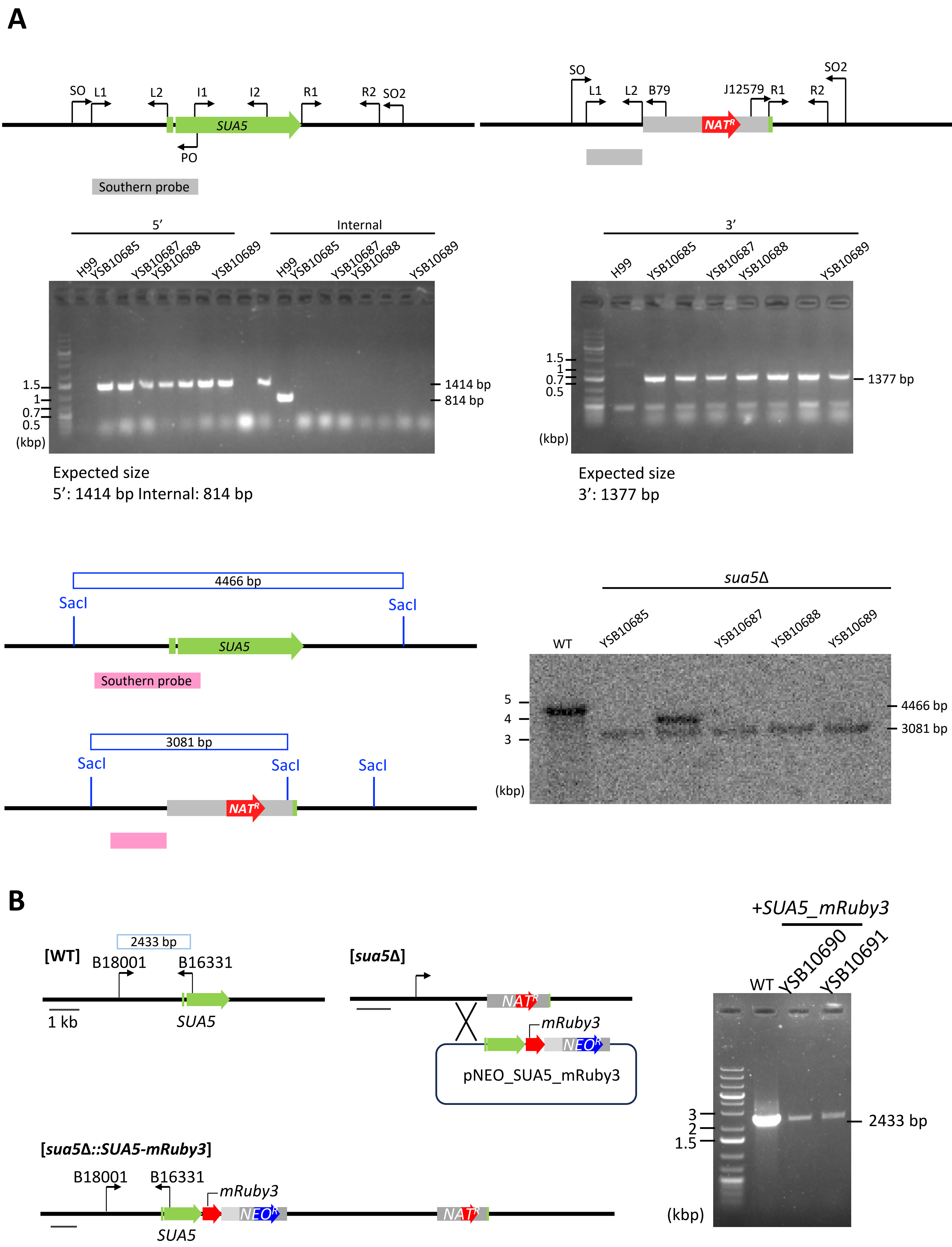

Fig S1. Construction and verification of *SUA5* deleted and complemented strains.

(A) Genotypic analysis of *sua5*Δ mutants. The upper panel depicts the gene disruption strategy where the *SUA5* gene is replaced with the *NAT* selection marker. The middle panel presents diagnostic PCR results, confirming 5′-end and 3′-end recombinations as well as internal deletion of the *SUA5* gene. For Southern blot verification, genomic DNA from wild-type (H99S) and *sua5*Δ mutants (YSB10685, YSB10687, YSB10688, and YSB10689) was digested with SacI. (B) Construction of *SUA5* complemented strains. For generating mRuby3-tagged strains, the native *SUA5* promoter and ORF were subcloned into the pNEO_mRuby3 vector. The plasmid was linearized using SalI and introduced into the *sua5*Δ mutant (YSB10685) through biolistic transformation. Targeted insertion was validated by diagnostic PCR using primer pairs (B18001 and B16331).
