# Supplementary material for: Sua5 catalyzing universal t6A tRNA modification is responsible for multifaceted functions of the KEOPS complex in Cryptococcus neoformans
Source: mSphere. 2023 Dec 12;9(1):e00557-23. doi: 10.1128/msphere.00557-23 (PMC10826353; doi:10.1128/msphere.00557-23)
Supplement: Fig. S2 — Monitoring of the cell cycle in the SUA5 mutant via flow cytometry. [file msphere.00557-23-s0002.pdf]

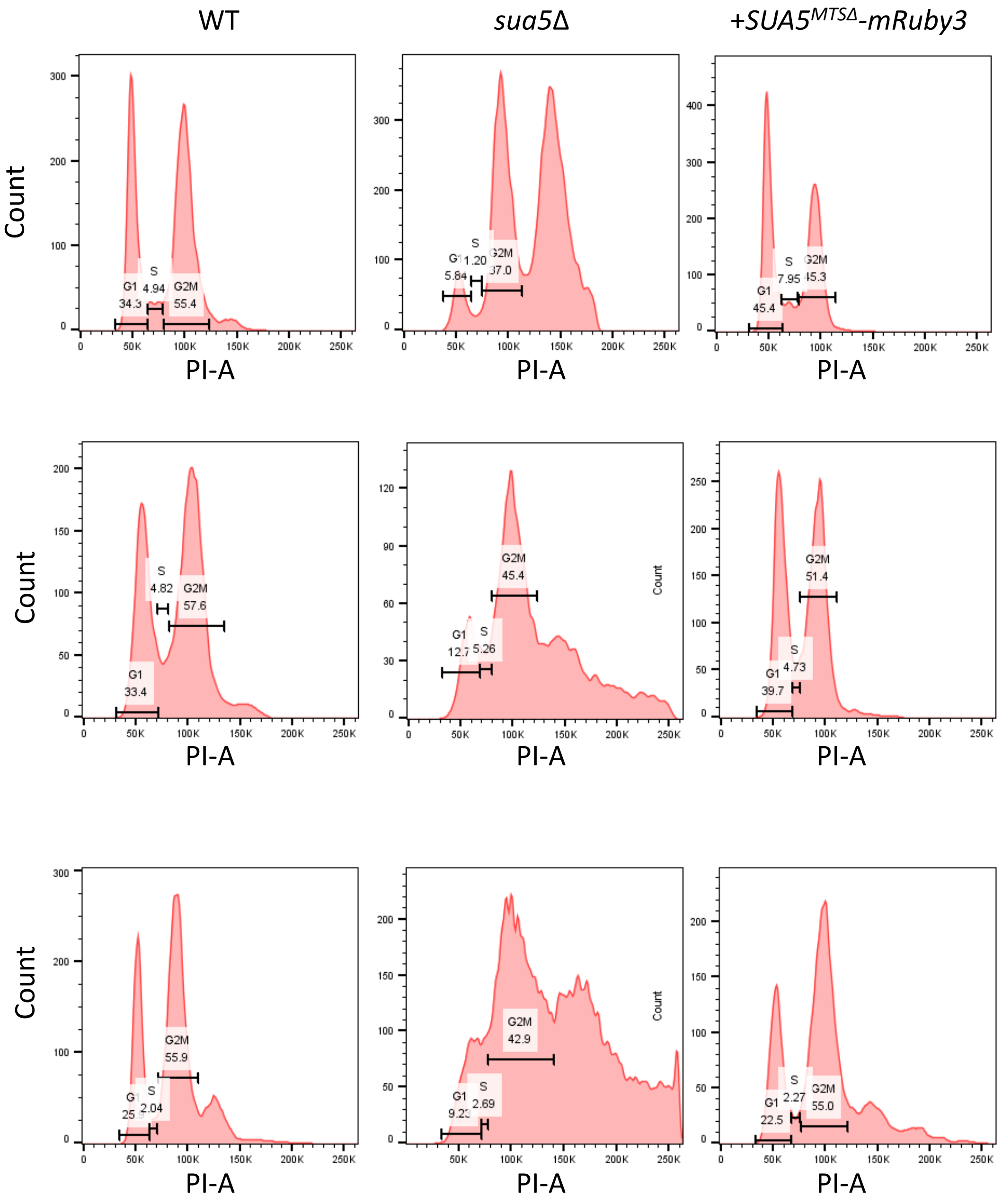


**Fig S2. Monitoring of the cell cycle in the *SUA5* mutant via flow cytometry.**

Cells were stained with propidium iodide (PI), with 10,000 cells analyzed per sample. The presented figures, arranged from top to bottom, correspond to three biological replicates. Within each graph, the distribution percentages for the cell cycle are indicated by G1, S, and G2/M DNA content levels.
