# Supplementary material for: Sua5 catalyzing universal t6A tRNA modification is responsible for multifaceted functions of the KEOPS complex in Cryptococcus neoformans
Source: mSphere. 2023 Dec 12;9(1):e00557-23. doi: 10.1128/msphere.00557-23 (PMC10826353; doi:10.1128/msphere.00557-23)
Supplement: Fig. S3 — Visualization of capsule formation in SUA5 and KEOPS mutants. [file msphere.00557-23-s0003.pdf]

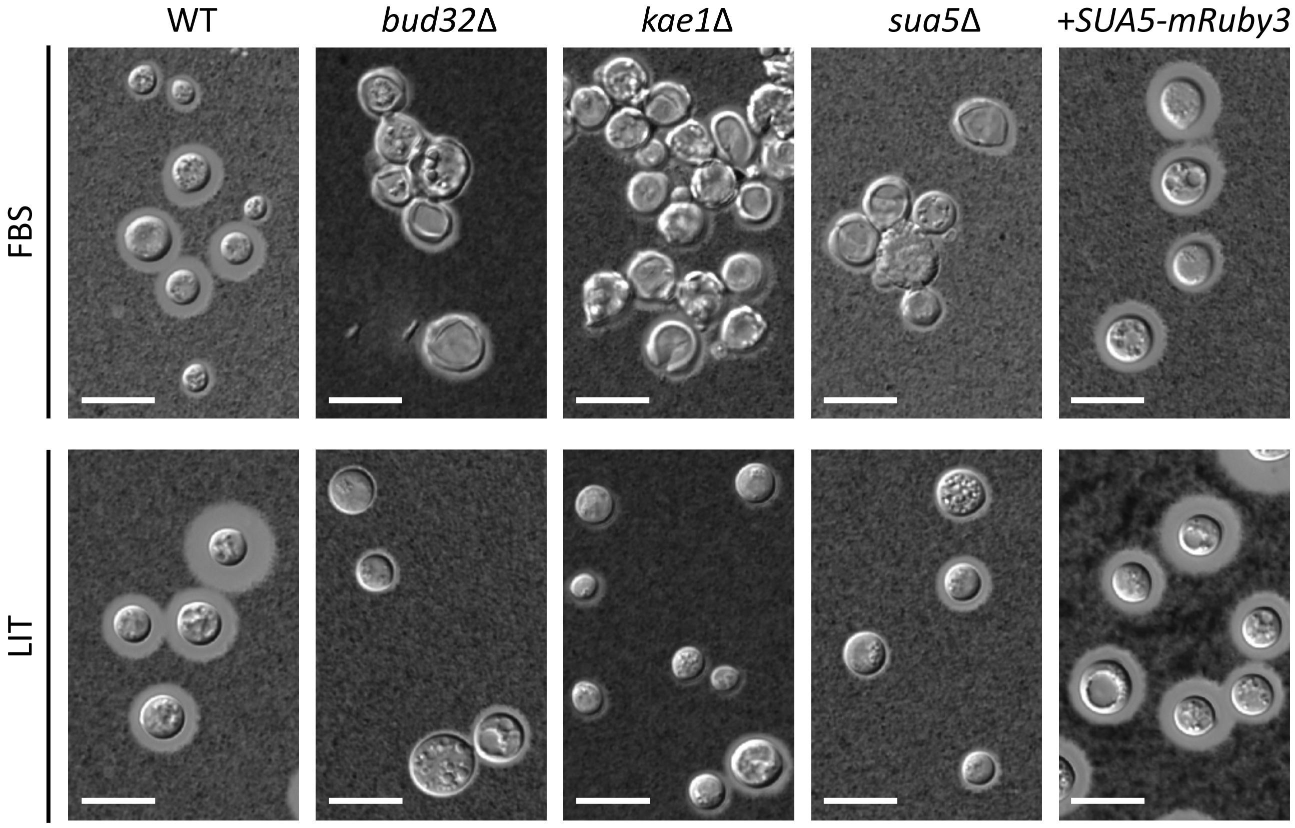

Fig S3. Visualization of capsule formation in *SUA5* and KEOPS mutants.

Strains including wild-type (H99S) *kae1*Δ (YSB4863), *bud32*Δ (YSB1968), *sua5*Δ (YSB10685), and *sua5*Δ::*SUA5-mRuby3* (YSB10690) were grown in either fetal bovine serum (FBS) or Littman (LIT) capsule-inducing solid media for two days at 37°C. Capsule formation was assessed post-incubation. After scraping, strains were mixed with India ink and observed under DIC microscopy. Scale bar = 10 µm.
