# Supplementary material for: Sua5 catalyzing universal t6A tRNA modification is responsible for multifaceted functions of the KEOPS complex in Cryptococcus neoformans
Source: mSphere. 2023 Dec 12;9(1):e00557-23. doi: 10.1128/msphere.00557-23 (PMC10826353; doi:10.1128/msphere.00557-23)
Supplement: Fig. S4 — Construction of C. neoformans strains lacking the mitochondria targeting sequence (MTS) in the SUA5 allele. [file msphere.00557-23-s0004.pdf]

Figure S4. (Choi et al., 2023)

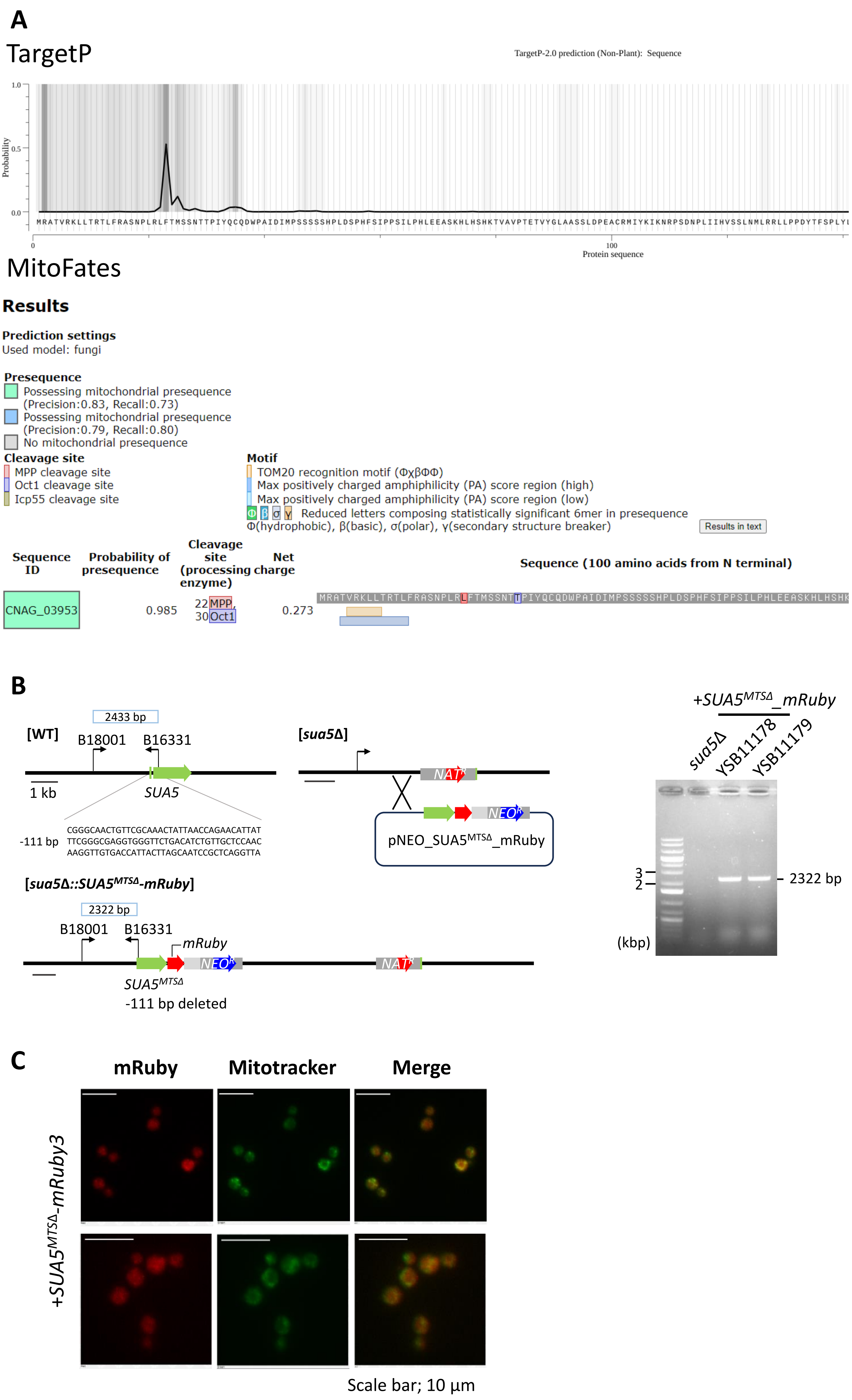

Fig S4. Construction of *C. neoformans* strains lacking the mitochondria targeting sequence (MTS) in the *SUA5* allele.

(A) MTS prediction. The mitochondria targeting sequence was identified using TargetP and MitoFates software, predicting the N-terminal 22 amino acids as essential for mitochondrial localization. (B) Construction of *sua5*Δ::*SUA5*$^{MTS\Delta}$-*mRuby3* strains. A 111-bp-deleted *SUA5* allele, which includes its promoter, was cloned into the pNEO_mRuby3 vector (YSB11178 and YSB11179). The plasmid was linearized with SalI introduced into *sua5*Δ mutant (YSB10685) via biolistic transformation. Diagnostic PCR confirmed targeted integration. (C) Localization of Sua5$^{MTS\Delta}$-mRuby3. Cells expressing *SUA5*$^{MTS\Delta}$-*mRuby3* (YSB11178) were fixed and stained with Mitotracker to visualize the mitochondrial localization. Scale bar = 10 µm.
