# Supplementary material for: Sua5 catalyzing universal t6A tRNA modification is responsible for multifaceted functions of the KEOPS complex in Cryptococcus neoformans
Source: mSphere. 2023 Dec 12;9(1):e00557-23. doi: 10.1128/msphere.00557-23 (PMC10826353; doi:10.1128/msphere.00557-23)
Supplement: Fig. S5 — Construction of sua5Δ bud32Δ double mutants. [file msphere.00557-23-s0005.pdf]

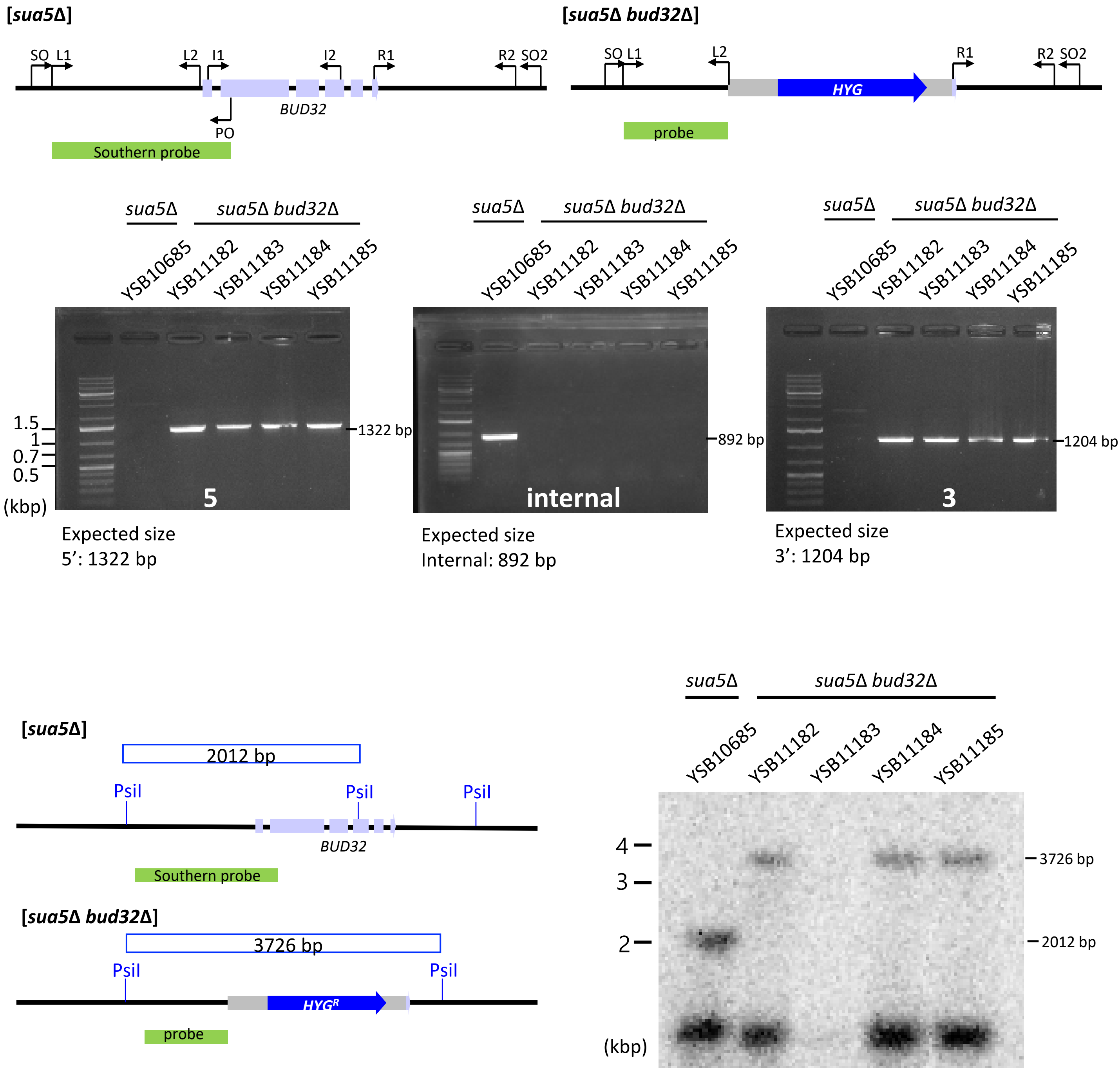

Fig S5. Construction of *sua5*Δ *bud32*Δ double mutants.

The upper panel outlines the gene disruption strategy, where the *BUD32* gene is replaced with a deletion cassette carrying the *HYG* selection marker in the *sua5*Δ. The middle panel displays the results of diagnostic PCR to verify 5′-end and 3′-end recombinations as well as internal deletion of the *BUD32* gene. The bottom panel showcases the Southern blot analysis performed on *sua5*Δ and *sua5*Δ *bud32*Δ mutants. Genomic DNA from *sua5*Δ (YSB10685) and *sua5*Δ *bud32*Δ (YSB11182, YSB11183, YSB11184, and YSB11185) mutants was digested with PsiI.
