# Supplementary material for: Sua5 catalyzing universal t6A tRNA modification is responsible for multifaceted functions of the KEOPS complex in Cryptococcus neoformans
Source: mSphere. 2023 Dec 12;9(1):e00557-23. doi: 10.1128/msphere.00557-23 (PMC10826353; doi:10.1128/msphere.00557-23)
Supplement: Fig. S6 — Construction of qri7Δ mutants. [file msphere.00557-23-s0006.pptx]

## Slide 1
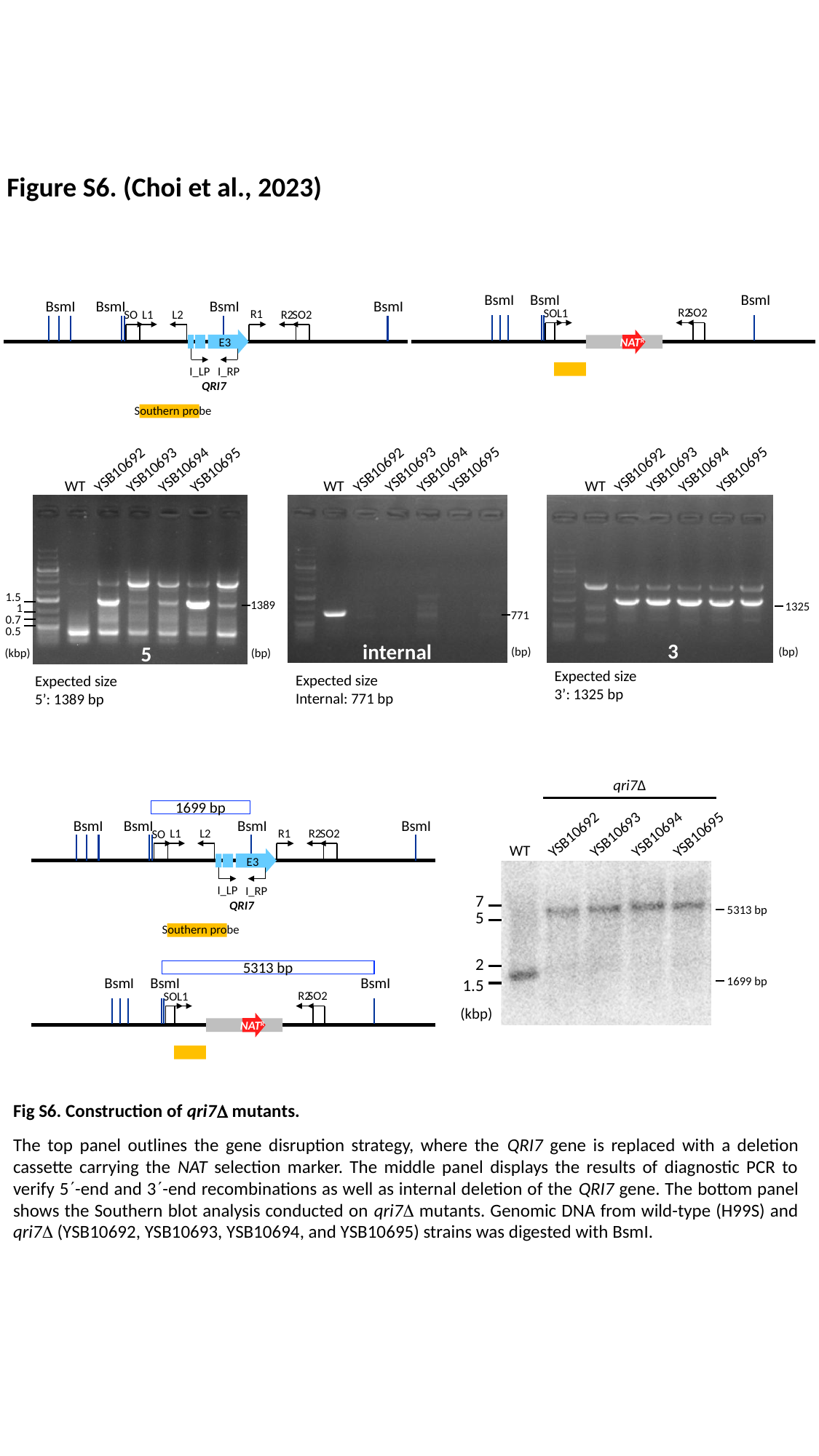

Figure S6. (Choi et al., 2023)
BsmI
BsmI
BsmI
R2
SO2
L1
SO
NATR
BsmI
BsmI
BsmI
BsmI
R1
R2
SO2
L1
L2
SO
E3
I_LP
I_RP
QRI7
Southern probe
YSB10692
YSB10693
YSB10694
YSB10695
YSB10692
YSB10693
YSB10694
YSB10695
YSB10692
YSB10693
YSB10694
YSB10695
WT
WT
WT
1.5
1389
1325
1
771
0.7
0.5
3’
internal
5’
(bp)
(bp)
(kbp)
(bp)
Expected size
3’: 1325 bp
Expected size
Internal: 771 bp
Expected size
5’: 1389 bp
qri7∆
1699 bp
BsmI
BsmI
BsmI
BsmI
R1
R2
SO2
L1
L2
SO
E3
I_LP
I_RP
QRI7
Southern probe
YSB10692
YSB10693
YSB10694
YSB10695
WT
7
5313 bp
5
2
5313 bp
BsmI
BsmI
BsmI
R2
SO2
L1
SO
NATR
1699 bp
1.5
(kbp)
Fig S6. Construction of qri7Δ mutants.
The top panel outlines the gene disruption strategy, where the QRI7 gene is replaced with a deletion cassette carrying the NAT selection marker. The middle panel displays the results of diagnostic PCR to verify 5′-end and 3′-end recombinations as well as internal deletion of the QRI7 gene. The bottom panel shows the Southern blot analysis conducted on qri7Δ mutants. Genomic DNA from wild-type (H99S) and qri7Δ (YSB10692, YSB10693, YSB10694, and YSB10695) strains was digested with BsmI.
