# Supplementary material for: Sua5 catalyzing universal t6A tRNA modification is responsible for multifaceted functions of the KEOPS complex in Cryptococcus neoformans
Source: mSphere. 2023 Dec 12;9(1):e00557-23. doi: 10.1128/msphere.00557-23 (PMC10826353; doi:10.1128/msphere.00557-23)
Supplement: Fig. S7 — The role of Sua5 in C. neoformans growth on non-fermentable carbon sources. [file msphere.00557-23-s0007.pdf]

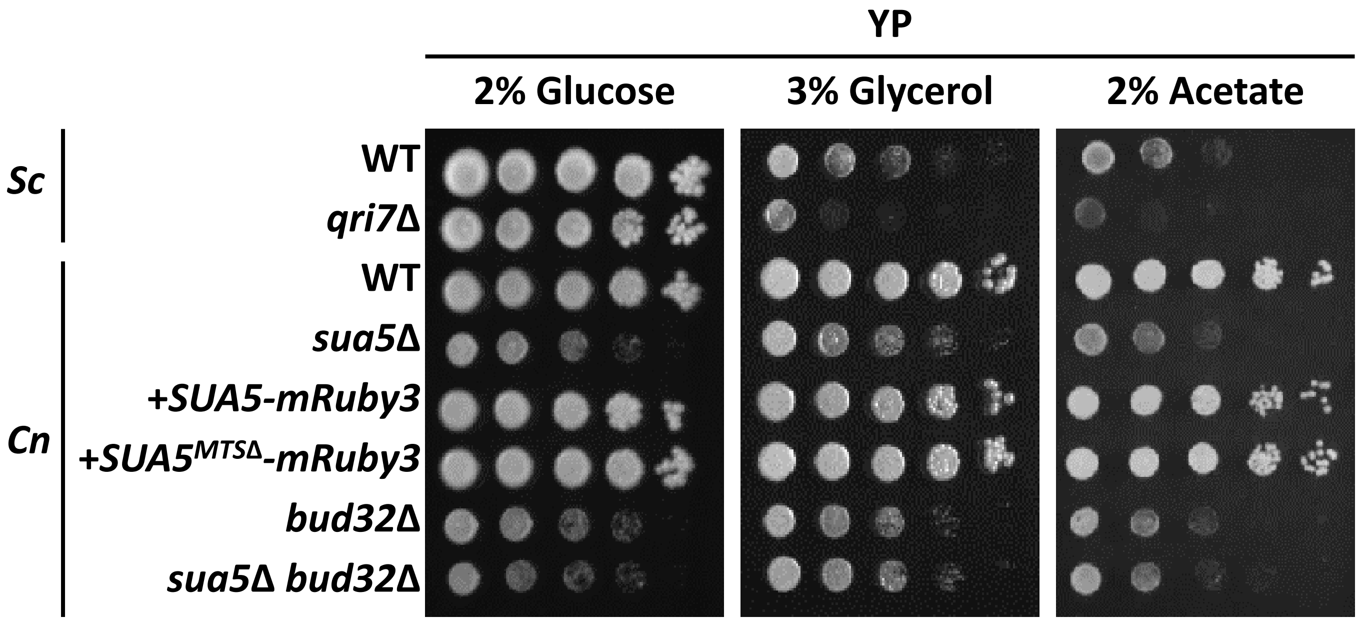

Fig S7. The function of Sua5 in non-fermentable carbon source growth.

The wild-type strains of *S. cerevisiae* (*Sc*) (BY4742), *Sc qri7*Δ (138-F-3), *C. neoformans* (*Cn*) (H99S), *sua5*Δ (YSB10685), +*SUA5-mRuby3* (YSB10690), +*SUA5^{MTSΔ}-mRuby3* (YSB11178), *bud32*Δ (YSB1968), and *sua5*Δ *bud32*Δ (YSB11182) were cultured in YPD broth at 30°C, serially diluted (1 to 10^4), and spotted onto YP containing various carbon sources (2% glucose, 3% glycerol, or 2% acetate), For 4 days, the plates were cultured at 30°C.
