# Supplementary material for: Sua5 catalyzing universal t6A tRNA modification is responsible for multifaceted functions of the KEOPS complex in Cryptococcus neoformans
Source: mSphere. 2023 Dec 12;9(1):e00557-23. doi: 10.1128/msphere.00557-23 (PMC10826353; doi:10.1128/msphere.00557-23)
Supplement: Table S1 — List of strains used in this study. [file msphere.00557-23-s0008.pdf]

Table S1. List of strains used in this study

Strains	Genotype	Parents	Reference
	C. neoformans		
H99	*MATα*		(1)
KN99	*MAT***a**		(2)
YSB1968	*MATα bud32Δ::NAT-STM#296*	H99	(3), (4)
YSB4863	*MATα kae1Δ::NAT-STM#212*	H99	(4)
YSB10685	*MATα sua5Δ::NAT-STM#6*	H99	This study
YSB10686	*MATα sua5Δ::NAT-STM#6*	H99	This study
YSB10687	*MATα sua5Δ::NAT-STM#6*	H99	This study
YSB10690	*MATα sua5Δ::SUA5-mRuby3-NEO*	YSB10685	This study
YSB10691	*MATα sua5Δ::SUA5-mRuby3-NEO*	YSB10685	This study
YSB11178	*MATα sua5Δ::*$SUA5^{MTS\Delta}$*-mRuby3-NEO*	YSB10685	This study
YSB11179	*MATα sua5Δ::*$SUA5^{MTS\Delta}$*-mRuby3-NEO*	YSB10685	This study
YSB11182	*MATα sua5Δ::NAT-STM#6 bud32Δ::HYG*	YSB10685	This study
YSB11183	*MATα sua5Δ::NAT-STM#6 bud32Δ::HYG*	YSB10685	This study
YSB11184	*MATα sua5Δ::NAT-STM#6 bud32Δ::HYG*	YSB10685	This study
YSB10692	*MATα qri7Δ::NAT-STM#119*	H99	This study
YSB10693	*MATα qri7Δ::NAT-STM#119*	H99	This study
YSB10694	*MATα qri7Δ::NAT-STM#119*	H99	This study
	S. cerevisiae		
BY4742	*MATα his3Δ1 leu2Δ0 lys2Δ0 ura3Δ0*		(5)
138-F-3	*MATα qri7Δ::KanMX*	BY4742	YSC1056 (Yeast homozygous diploid collection)

1. Perfect JR, Ketabchi N, Cox GM, Ingram CW, Beiser CL. 1993. Karyotyping of *Cryptococcus neoformans* as an epidemiological tool. J Clin Microbiol 31:3305-9.
2. Nielsen K, Cox GM, Wang P, Toffaletti DL, Perfect JR, Heitman J. 2003. Sexual cycle of *Cryptococcus neoformans* var. *grubii* and virulence of congenic a and alpha isolates. Infect Immun 71:4831-41.
3. Lee KT, So YS, Yang DH, Jung KW, Choi J, Lee DG, Kwon H, Jang J, Wang LL, Cha S, Meyers GL, Jeong E, Jin JH, Lee Y, Hong J, Bang S, Ji JH, Park G, Byun HJ, Park SW, Park YM, Adedoyin G, Kim T, Averette AF, Choi JS, Heitman J, Cheong E, Lee YH, Bahn YS. 2016. Systematic functional analysis of kinases in the fungal pathogen *Cryptococcus neoformans*. Nat Commun 7:12766.
4. Choi Y, Jeong E, Lee DG, Jin JH, So YS, Yu SR, Lee KJ, Ha Y, Lin CJ, Chen YL, Park JB, Cho HS, Averette AF, Heitman J, Lee KH, Lee K, Bahn YS. 2022. Unraveling the pathobiological role of the fungal KEOPS complex in *Cryptococcus neoformans*. mBio 13:e0294422.
5. Brachmann CB, Davies A, Cost GJ, Caputo E, Li J, Hieter P, Boeke JD. 1998. Designer deletion strains derived from *Saccharomyces cerevisiae* S288C: a useful set of strains and plasmids for PCR-mediated gene disruption and other applications. Yeast 14:115-32.
