# Supplementary material for: Sua5 catalyzing universal t6A tRNA modification is responsible for multifaceted functions of the KEOPS complex in Cryptococcus neoformans
Source: mSphere. 2023 Dec 12;9(1):e00557-23. doi: 10.1128/msphere.00557-23 (PMC10826353; doi:10.1128/msphere.00557-23)
Supplement: Table S2 — List of primers used in this study. [file msphere.00557-23-s0009.pdf]

Table S2. List of primers used in this study

Name	Primer description	Sequence (5' to 3')
B16326	CNAG_03953 (SUA5) L1	GAGTGCATGAAGCTGGCATA
B16327	CNAG_03953 (SUA5) L2	GCTCACTGGCCGTCGTTTTACGCTTTTGGCCTTACTGTGCT
B16328	CNAG_03953 (SUA5) R1	CATGGTCATAGCTGTTTCCTGGGAAACAAGTTCTGGGTGGA
B16329	CNAG_03953 (SUA5) R2	GTCTTTCTTTGGTGGCCGTA
B16330	CNAG_03953 (SUA5) SO	AAAAGCCAAACCAACGACAC
B16331	CNAG_03953 (SUA5) PO	CGAAGCTGCAAGACCATACA
B16506	SUA5 knock out internal_LP	TGTATGGTCTTGCAGCTTCG
B16507	SUA5 knock out internal_RP	GCAGATGCAGATGATGGAGA
B16332	CNAG_05969 (QRI7) L1	CGTTGTCTGATGGCAAGAGT
B16333	CNAG_05969 (QRI7) L2	GCTCACTGGCCGTCGTTTTACGTATAGGCGACGGCATCAAT
B16334	CNAG_05969 (QRI7) R1	CATGGTCATAGCTGTTTCCTGCAGGCGGGATGCAATGTA
B16335	CNAG_05969 (QRI7) R2	TGGGATTAGGACATGTCTGTTG
B16336	CNAG_05969 (QRI7) SO	CCTGTAGGCAGCCTGTAAGC
B16337	CNAG_05969 (QRI7) PO	AGGATGAGGAACGGGAATTT
B17994	CNAG_05969 (QRI7) SO2	TTTAGGAAATTCTGAAACAAGCTC
B18400	CNAG_05969 (QRI7) internal	AGGGGTGCATCATATGGTGT
B18401	CNAG_05969 (QRI7) internal	GCGGTGGGTAGTAGAGGTTT
B17863	pNEO_SUA5_mRuby3 FWD	ctctagatgcatgctcgagc*ggccgc*GTATAAACTACGAAGGCC
B17864	pNEO_SUA5_mRuby3 REV	atagagccaccgccacctgc*ggccgc*CGTAGAACTAACGTCCAC
B17940	pNEO_SUA5_mRuby3 seq 1	AGAGCTCGTCCTGTTAACTAT
B17941	pNEO_SUA5_mRuby3 seq 2	AATCGGATGTCGGAACGA
B17942	pNEO_SUA5_mRuby3 seq 3	TTATGCCTTCTTCCTCATCC
B17943	pNEO_SUA5_mRuby3 seq 4	GAAGGAGGCATCATTTAGGC
B18001	SUA5 CSO	GGAACCAAAGCGTCCAAATA
B18833	Sua5 mitochondria-targeted	GCCAAAAGCCATAGGGGCATGTTCACAATGTCGAGCAAC
B18834	Sua5 mitochondria-targeted	GTTGCTCGACATTGTGAACATGCCCCTATGGCTTTTGGC
B18889	CNAG_02712 L1 (BUD32)	ATAGGGGATGACCTTGGAG
B18890	CNAG_02712 L2 (BUD32)	CACTCGAATCCTGCATGCTGATGCCAAAGACCAGTG
B18891	CNAG_02712 R1 (BUD32)	TCAGGATCTTCATGGCTCCGAGAAGAGGAAGGAAGAGAGAC
B18892	CNAG_02712 R2 (BUD32)	GAGCGATAATAGCCACCAC
B18893	CNAG_02712 SO (BUD32)	GGGCAATCTTTCTTCGTC
B18894	CNAG_02712 PO (BUD32)	CTCGTTCTCTGGTTCTTCTG
B18895	CNAG_02712 SO2 (BUD32)	ACTTCACGCCTCATTTGCTT
B18896	CNAG_02712 internal_LP (BUD32)	CCTCTACTTAGCCGGGGAAC
B18897	CNAG_02712 internal_RP (BUD32)	GGATGCGTAGAAGCAAAAGC
B20971	BUD32 southern probe PO2	GCTAATTGTCGCAGGTCCTC
